# Stabilized Cubic GeTe With Matched Grain‐Boundary Networks and Band Convergence for High‐Performance Dual‐Mode Thermoelectric Devices

**DOI:** 10.1002/advs.76668

**Published:** 2026-07-17

**Authors:** Xiaobo Tan, Xuri Rao, Huangshui Ma, Jiaxing Luo, Fan Feng, Zijian Lin, Ruiheng Li, Maoji Tian, Siqi Huo, Min Hong, Ran Ang

**Affiliations:** ^1^ Key Laboratory of Radiation Physics and Technology Institute of Nuclear Science and Technology Ministry of Education Sichuan University Chengdu China; ^2^ Centre for Future Materials Springfield Campus University of Southern Queensland Queensland Australia; ^3^ College of Physics Sichuan University Chengdu China; ^4^ Institute of New Energy and Low‐Carbon Technology Sichuan University Chengdu China

**Keywords:** thermoelectrics, GeTe, cubic phase, grain boundary, cooling, power generation

## Abstract

Lead‐free GeTe‐based thermoelectrics exhibit strong potential; however, device performance is limited by the intrinsic ferroelectric phase transition and structural instability. Here, we propose a cooperative structural‐electronic regulation strategy that stabilizes the cubic phase and simultaneously enhances the thermoelectric and mechanical properties. AgSbTe_2_ alloying completely suppresses the ferroelectric transition over the entire operating temperature range, while complementary I doping produces refined single‐phase domains with well‐matched multi‐degree‐of‐freedom grain boundary networks, together with a precisely tuned band structure. These synergistic effects intensify phonon scattering, optimize carrier transport, and increase the effective mass, thereby elevating the power factor and substantially reducing the lattice thermal conductivity (*κ*_L_), thereby significantly improving the weighted mobility (*µ*_w_)‐to‐*κ*_L_ ratio. The optimized composition achieves a room‐temperature *zT* of ∼0.7 and a peak *zT* of ∼2.2 at 773 K, demonstrating superior performance from near‐ambient to intermediate temperatures. Concurrently, grain refinement effectively enhances mechanical integrity, yielding a compressive strength of ∼248 MPa. A 7‐pair power‐generation device delivers ∼11% efficiency at Δ*T* = 470 K, while a cooling device achieves a maximum Δ*T*_max_ of ∼65.6 K at 350 K. This study establishes a generalizable framework for concurrent phase stabilization and performance optimization, enabling scalable, high‐reliability GeTe‐based dual‐mode thermoelectric devices.

## Graphical abstract

I‐doping stabilizes the cubic phase of GeTe and generates “electron‐transparent, phonon‐blocking” grain boundary networks while inducing valence‐band convergence. This synergistic structural‐electronic design enables effective electron‐phonon decoupling, achieving an exceptional average *zT*_ave_ of ∼1.62, a peak *zT* of ∼2.2, and enhanced mechanical robustness (∼248 MPa). These advances further realize high‐reliability dual‐mode functionality, delivering 11% power‐generation efficiency and a 65.6 K cooling temperature difference.

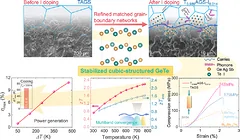

## 1 Introduction

Thermoelectric (TE) technology, which enables direct solid‐state conversion between heat and electricity, has garnered extensive attention in energy and materials research owing to its broad prospects in waste heat harvesting, power generation under harsh environments, and solid‐state cooling [1, 2, 3, 4, 5]. The practical deployment of TE devices across these scenarios critically relies on TE materials that can simultaneously deliver high conversion efficiency and long‐term operational stability [6, 7, 8]. Thus, advancing the intrinsic performance and structural reliability of TE materials remains a central challenge in the field.

The energy‐conversion capability of TE materials is quantified by the dimensionless figure of merit (*zT*), *zT* = *S* ^2^ *σT*/*κ*, in which *S* is the Seebeck coefficient, *σ* is the electrical conductivity, *κ* is the thermal conductivity consisting of lattice (*κ*_L_) and electronic (*κ*_e_) components, and *T* is the absolute temperature. Achieving a high *zT* requires a rare combination of a large *S*, high *σ*, and a suppressed *κ*. However, these transport parameters are inherently interdependent because they originate from the same electronic structure and scattering mechanisms [9, 10, 11, 12]. Specifically, strategies that effectively suppress *κ*_L_, such as introducing multi‐scale defect structures, alloy scattering, or hierarchical microstructures, often introduce additional carrier scattering, thereby deteriorating carrier mobility (*µ*_H_) and limiting further enhancement of the power factor (*PF* = *S* ^2^ *σ*) [13, 14, 15, 16]. Conversely, electronic‐band engineering aimed at optimizing carrier transport may inadvertently reduce phonon scattering strength, compromising thermal management.

In practical TE applications, constructing high‐performance TE devices capable of operating across broad temperature windows—from near‐room‐temperature cooling to mid‐ and high‐temperature power generation—requires not only excellent peak *zT* but also a high average *zT_ave_*, which more directly determines the actual energy conversion output of devices [11, 17, 18]. Beyond efficiency, the long‐term reliability of TE devices critically depends on their mechanical robustness, including compressive strength, Vickers hardness, and thermal expansion coefficient (CTE) [19, 20, 21]. These mechanical attributes govern the structural integrity of devices under thermal cycling, large temperature gradients, and mechanical stresses, ultimately defining the service lifetime and environmental adaptability of TE devices. Therefore, simultaneously enhancing both TE performance and mechanical durability is essential for enabling the real‐world deployment of advanced TE systems.

GeTe, a representative IV‐VI multi‐valley degenerate semiconductor, has emerged as a leading high‐temperature, lead‐free TE material. Under ambient conditions, GeTe crystallizes in a rhombohedral ferroelectric phase (*R3m*). Upon heating to 700 K, it undergoes a ferroelectric‐to‐paraelectric transition into a high‐symmetry cubic phase (*Fm3̅m*) [22, 23]. This transition increases the interaxial angle from 88.2° to 90.0°, and critically, reverses the energetic ordering of the light and heavy valence bands, driving a temperature‐induced band convergence that benefits high‐temperature transport [24, 25, 26]. However, despite significant progress in composition and microstructure engineering, the intrinsic ferroelectric phase transition remains the most fundamental bottleneck hindering the practical device integration of GeTe‐based materials.

In highly integrated devices, repeated phase‐transition‐induced volume and symmetry changes generate cumulative strain and intergranular stress during operation [27, 28, 29]. This strain accumulation progressively degrades electrical transport, introduces microcrack formation, and accelerates mechanical fatigue, eventually jeopardizing device stability and even triggering catastrophic structural failure. Such transition‐driven instability has long been a primary obstacle preventing GeTe from achieving the reliability required for large‐scale TE deployment.

The pseudo‐binary (GeTe)*_x_* (AgSbTe_2_)_1‐_ *_x_* system has emerged as an effective platform for overcoming the intrinsic ferroelectric phase transition of GeTe, a long‐standing bottleneck that triggers band splitting, structural instability, and nonlinear thermal expansion [30, 31, 32]. By tailoring the AgSbTe_2_ content, the GeTe lattice can be continuously modulated from a rhombohedral to a cubic phase, enabling precise control over the ferroelectric distortion amplitude [33]. Notably, all synthesized compositions within the range *x* = 50–75 maintain a stabilized cubic structure over 323–773 K, fully suppressing the temperature‐driven ferroelectric transition and its associated anharmonicity [31, 34]. Both theoretical predictions and experimental data indicate that (GeTe)*_x_* (AgSbTe_2_)_1‐_ *_x_* behaves as a slightly degenerate semiconductor with an increased *S*, thereby delivering a strengthened *PF* [35, 36]. In parallel, the introduction of multiple atomic species into the cation sublattice produces substantial mass and strain fluctuations, while Ag‐rich nanoscale domains that intrinsically form at *x*≥15 serve as efficient mid‐ and long‐wavelength phonon scattering centers [37, 38, 39, 40]. These nanodomains not only suppress *κ*_L_ but also impede dislocation motion, conferring improved mechanical robustness—an increasingly critical requirement for device‐level integration, where devices endure significant thermomechanical stress [41].

Despite these advantages, a non‐negligible trade‐off persists: Ag‐rich secondary phases exhibit high resistivity, which severely degrades *µ*_H_ and *σ*, offsetting *PF* gains [34]. To mitigate this limitation, Zhang et al. employed phase‐diagram‐guided Ag/Sb non‐stoichiometry engineering (Ag_0.77_Sb_1.23_Te_2.23_), which eliminates secondary phases and restores *σ* [42]. However, the removal of nanoscale Ag‐rich domains simultaneously weakens phonon scattering, eroding the intrinsic advantage of *κ*_L_ suppression.

To overcome the persistent performance optimization bottlenecks of the (GeTe)*_x_* (AgSbTe_2_)_1‐_ *_x_* pseudo‐binary system, we propose a synergistic structural‐electronic regulation strategy. Building upon the phase‐diagram‐guided non‐stoichiometric composition Ag_0.77_Sb_1.23_Te_2.23_, we first stabilize a fully cubic, single‐phase matrix in (GeTe)_78_(Ag_0.77_Sb_1.23_Te_2.23_)_22_ [42]. Subsequently, we introduce iodine to substitute Te anions within this cubic phase, enabling simultaneous grain‐boundary engineering and electronic band optimization‐two key levers for achieving high‐efficiency and mechanically robust thermoelectrics.

As illustrated in Figure 1, I doping serves as an effective grain refiner that promotes the formation of a network of highly coherent and structurally matched grain boundaries (Figure 1a). Such well‐organized interfaces provide dual functionalities: they facilitate unobstructed electron transport to preserve *µ*_H_ while acting as effective phonon‐scattering barriers that suppress *κ*_L_. Concurrently, density functional theory (DFT) calculations reveal that iodine incorporation widens the band gap (*E*_g_) and induces converged multi‐valence‐band alignment at the valence band maximum (VBM) (Figure 1c). This modulation increases the density‐of‐states (DOS) effective mass (*m* ^*^) and elevates the transport parameter *µ*_w_/*κ*_L_ (with *µ*_w_ = *µ*(*m* ^*^/*m*_e_)^3/2^), ultimately enabling synergistic enhancement of the power factor and phonon scattering. This integrated strategy substantially boosts both the average *PF*_ave_ and phonon‐suppression capability, reducing *κ*_L_ to as low as 0.45 W m^−1^ K^−1^. As a result, the optimized material delivers a peak *zT* of ∼2.2 at 773 K and an exceptionally high average *zT*_ave_ of ∼1.62 across 300–773 K—placing it among the top‐performing cubic GeTe‐based thermoelectrics (Figure 1d).

**FIGURE 1:**
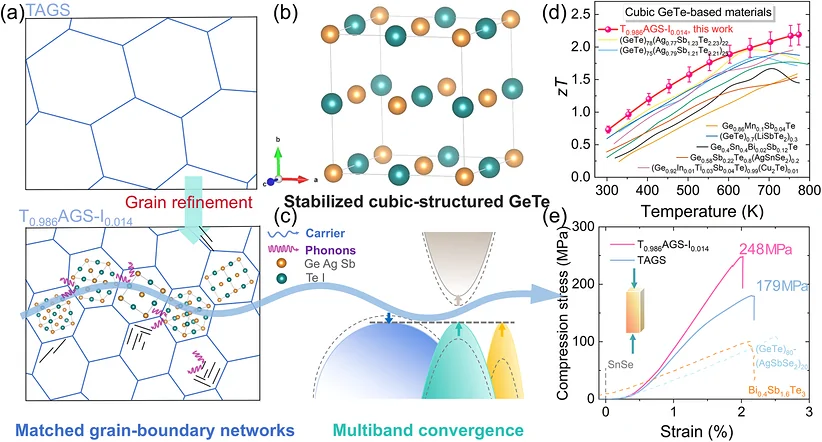
Synergistic structural‐electronic strategies for enhancing the thermoelectric and mechanical properties of I‐doped (GeTe)_78_(Ag_0.77_Sb_1.23_Te_2.23_)_22_ (denoted as TAGS)‐based materials. (a) Schematic illustration of the microstructural evolution induced by I doping, highlighting grain refinement, increased grain‐boundary density, formation of multi‐scale defect structures, and the resulting modulation of phonon scattering and charge transport behaviors in the TAGS matrix. (b) Schematic diagram of the stabilized cubic crystal structures obtained through AgSbTe_2_ alloying and subsequent I doping. (c) Depiction of the dynamic evolution of the electronic band structure, demonstrating band convergence and enhanced band degeneracy. (d) Comparison of temperature‐dependent *zT* of the T_0.986_AGS‐I_0.014_ sample with representative state‐of‐the‐art cubic‐phase GeTe materials [34, 42, 44, 45, 46, 47, 48]. (e) Comparison of compressive strength with previously reported high‐performance GeTe‐based systems, highlighting the significant mechanical robustness achieved in this work [49, 50].

Beyond transport optimization, the refined grain boundary architecture strengthens intergranular bonding and mitigates dislocation motion, yielding significant mechanical reinforcement. Vickers microhardness and compressive strength increase by ∼37% and ∼39%, respectively, compared to the undoped counterpart (Figure 1e). These improvements greatly enhance the machinability and durability of TE materials, endowing them with outstanding advantages for practical applications in micro functional devices [43]. This mechanical robustness, combined with superior TE efficiency, provides essential reliability for device‐level operation under thermal cycling and external mechanical loads. Benefiting from these dual enhancements, the material demonstrates excellent device performance. A single‐stage TE generator achieves a high‐power density of 2.03 W cm^−2^ under *ΔT* = 470 K, while a cooling device paired with *n*‐type Bi_2_Te_3_ attains a substantial maximum Δ*T*_max_ of ∼65.6 K under a hot‐side temperature of 350 K. These results significantly strengthen the technological prospects of GeTe‐based thermoelectrics for both solid‐state cooling and mid‐temperature waste‐heat harvesting.

## 2 Results and Discussion

The intrinsic rhombohedral‐to‐cubic structural phase transition of GeTe is known to induce pronounced nonlinear thermal expansion and large differential heat flow (Figure 2a), which ultimately undermines the mechanical and thermal stability of GeTe‐based TE devices. To eliminate this detrimental instability, a (GeTe)_78_(Ag_0.77_Sb_1.23_Te_2.23_)_22_‐alloyed matrix (hereafter denoted as TAGS) was designed by precisely tuning the Ag_0.77_Sb_1.23_Te_2.23_ ternary composition to fully suppress the ferroelectric distortion of GeTe. Building on this stabilized matrix, partial substitution of Te with I was further introduced, and a series of high‐density T_1‐_ *_x_* AGS‐I*_x_* (*x* = 0‐0.018, Table S1) samples were synthesized through high‐temperature melting, high‐energy ball milling (BM), and hot pressing (HP). This dual strategy—matrix pre‐stabilization plus halogen‐induced local structural regulation—is aimed at simultaneously improving the structural robustness and phonon‐scattering capability.

**FIGURE 2:**
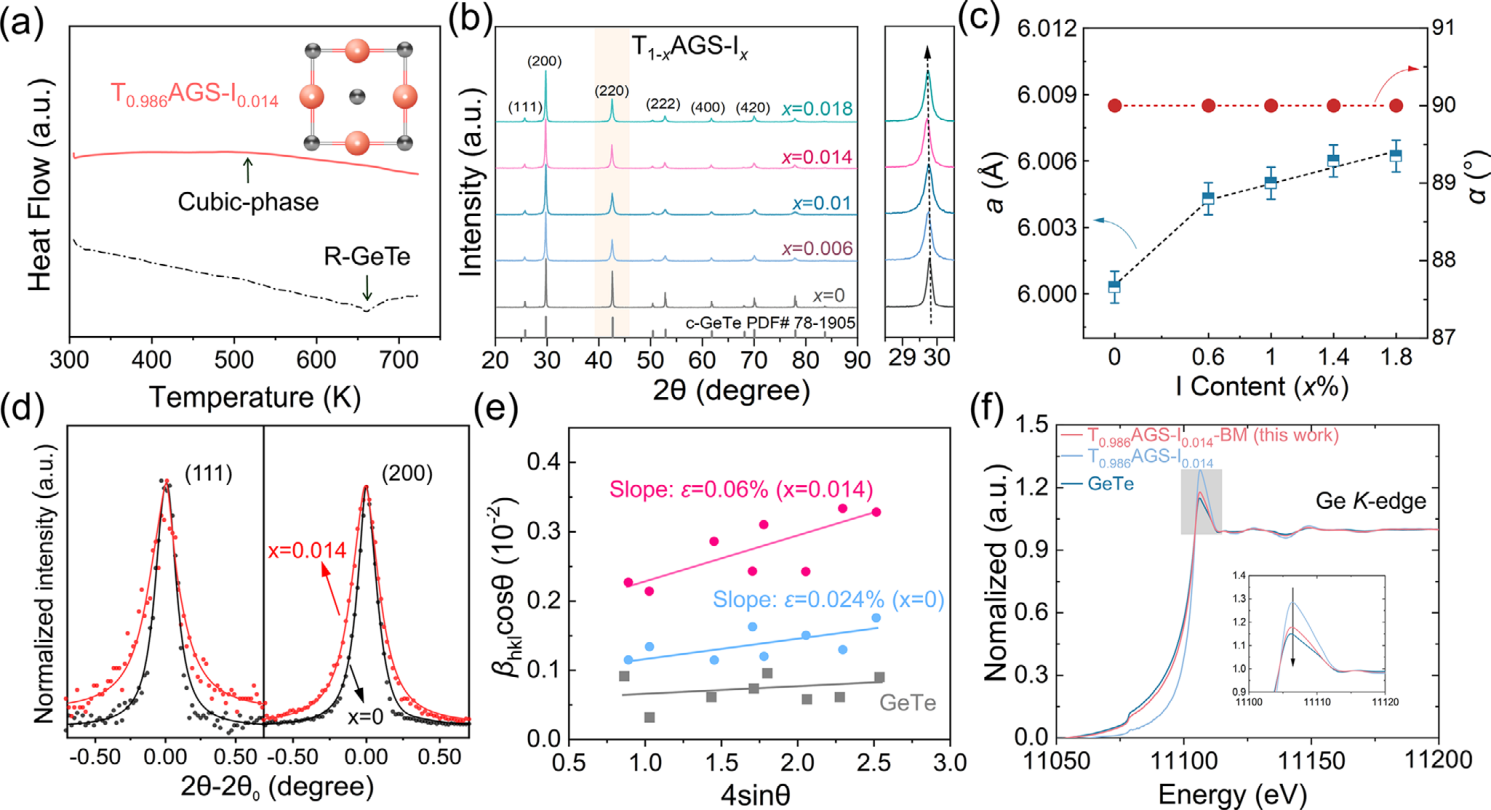
Phase structure and local structural characterizations of T_1‐_ *_x_* AGS‐I*_x_* (*x* = 0‐0.018) samples. (a) DSC curves of representative T_0.986_AGS‐I_0.014_ and pristine GeTe, showing the suppression of the ferroelectric phase transition. (b) Room‐temperature powder XRD patterns for T_1‐_ *_x_* AGS‐I*_x_* (*x* = 0‐0.018), confirming cubic phase formation across the series. (c) Lattice parameters and interaxial angles calculated from XRD refinements, illustrating the effect of AgSbTe_2_ alloying and I doping on unit‐cell stabilization. (d) Normalized (111) and (200) diffraction peaks for T_0.986_AGS‐I_0.014_ and TAGS samples, highlighting peak shifts and narrowing associated with lattice strain relaxation. (e) Lattice strain (*ε*) extracted from Williamson‐Hall analysis, revealing reduced microstrain in the I‐doped and ball‐milled samples. (f) Ge *K*‐edge XANES spectra of GeTe, T_0.986_AGS‐I_0.014_‐ingot, and T_0.986_AGS‐I_0.014_ ball‐milled (BM).

Powder X‐ray diffraction (XRD) patterns confirm that all samples crystallize into a single‐phase cubic GeTe structure (space group *Fm3̅m*), without any detectable secondary phases (Figure 2b). The absence of XRD peak splitting near 42°, together with differential scanning calorimetry (DSC) curves showing no thermal anomalies over the entire temperature range (Figure 2a), unambiguously demonstrates that this cubic phase remains stable throughout the full operating temperature range. This indicates that the combination of Ag‐Sb‐Te alloying and controlled I substitution effectively suppresses the structural phase transition that typically plagues pristine GeTe.

Despite the nearly identical ionic radii of I^−^ (2.2 Å) and Te^2−^ (2.21 Å), the diffraction peaks gradually shift toward lower angles with increasing I doping, revealing a gradual lattice expansion. Rietveld refinement confirms that all samples maintain a highly symmetric cubic lattice with fixed 90° lattice angles, whereas the lattice parameter displays a linear dependence on the I content (Figure 2c and Figure S1). This trend suggests that I incorporation may promote the re‐dissolution of Ge atoms into the TAGS matrix, counteracting the Ge vacancy tendency of GeTe, and in turn expands the average lattice volume. Compared with the small electronegativity difference (*Δχ*) between Te and Ge (*Δχ* = 0.09), the larger electronegativity difference between I and Ge (*Δχ* = 0.65) generates a stronger electron‐withdrawing effect around the Ge sites, providing a possible driving force for the re‐incorporation of Ge into the lattice. Subsequent quantitative SEM‐EDS analysis (Figure S5), which reveals an increased Ge content in the matrix after I doping, further confirms this point. Such defect chemistry evolution is expected to modulate both the carrier concentration (*n*_H_) and phonon dispersion.

To evaluate the lattice microstrain, Figure 2d compares the normalized (111) and (200) reflections for representative compositions. The T_1‐_ *_x_* AGS‐I*_x_* sample with *x* = 0.014 shows pronounced peak broadening relative to pristine TAGS, indicating enhanced strain accumulation triggered by atomic mass fluctuation and size mismatch. Quantitative Williamson‐Hall (W‐H) analysis further deconvolutes the full width at half maximum (FWHM) [51], revealing that the microstrain increases significantly from ∼0.024% in pristine TAGS to ∼0.06% in T_0.986_AGS‐I_0.014_ (Figure 2e). Such a substantial increase in microstrain reflects a strengthened short‐range disorder introduced synergistically by BM‐induced defect activation and I substitution [52].

As shown in Figure 2f, compared with pristine GeTe, the Ge K‐edge X‐ray absorption near‐edge structure (XANES) spectra of the optimized T_0.986_AGS‐I_0.014_ sample exhibit a pronounced change in white‐line intensity. These changes indicate that the synergistic regulation via AgSbTe_2_ alloying and I doping significantly alters the local chemical environment of Ge atoms, specifically manifested as increased local structural disorder and a more uniform electronic distribution around Ge sites. This result is mutually corroborated by the XRD microstrain analysis and the multi‐scale defect structures observed by High‐resolution TEM (HRTEM) [53].

The impact of these structural modifications on electronic transport is shown in Figure 3. All samples exhibit the characteristic behavior of a heavily doped degenerate semiconductor, with *σ* decreasing with temperature due to dominant acoustic phonon scattering in the low‐temperature regime. Above ∼600 K, *σ* increases slightly, which is attributed to thermally activated carrier concentration rather than bipolar conduction (Figure 3a). With increasing I content, *σ* exhibits a monotonic decrease, primarily arising from the modulation of *n*_H_. In contrast, *S* is substantially enhanced. At 300 K, *S* increases from ∼150 µV K^−1^ for pristine TAGS to ∼218 µV K^−1^ for *x* = 0.014, representing an impressive 40% improvement. The maximum *S* reaches ∼299 µV K^−1^ at 600 K (Figure 3b). As a result, the *PF* benefits from this optimized *σ‐S* balance, reaching ∼22.4 µW cm^−1^ K^−2^, at 300 K in T_0.986_AGS‐I_0.014_, compared with ∼16.7 µW cm^−1^ K^−2^ for pristine TAGS (Figure 3c).

**FIGURE 3:**
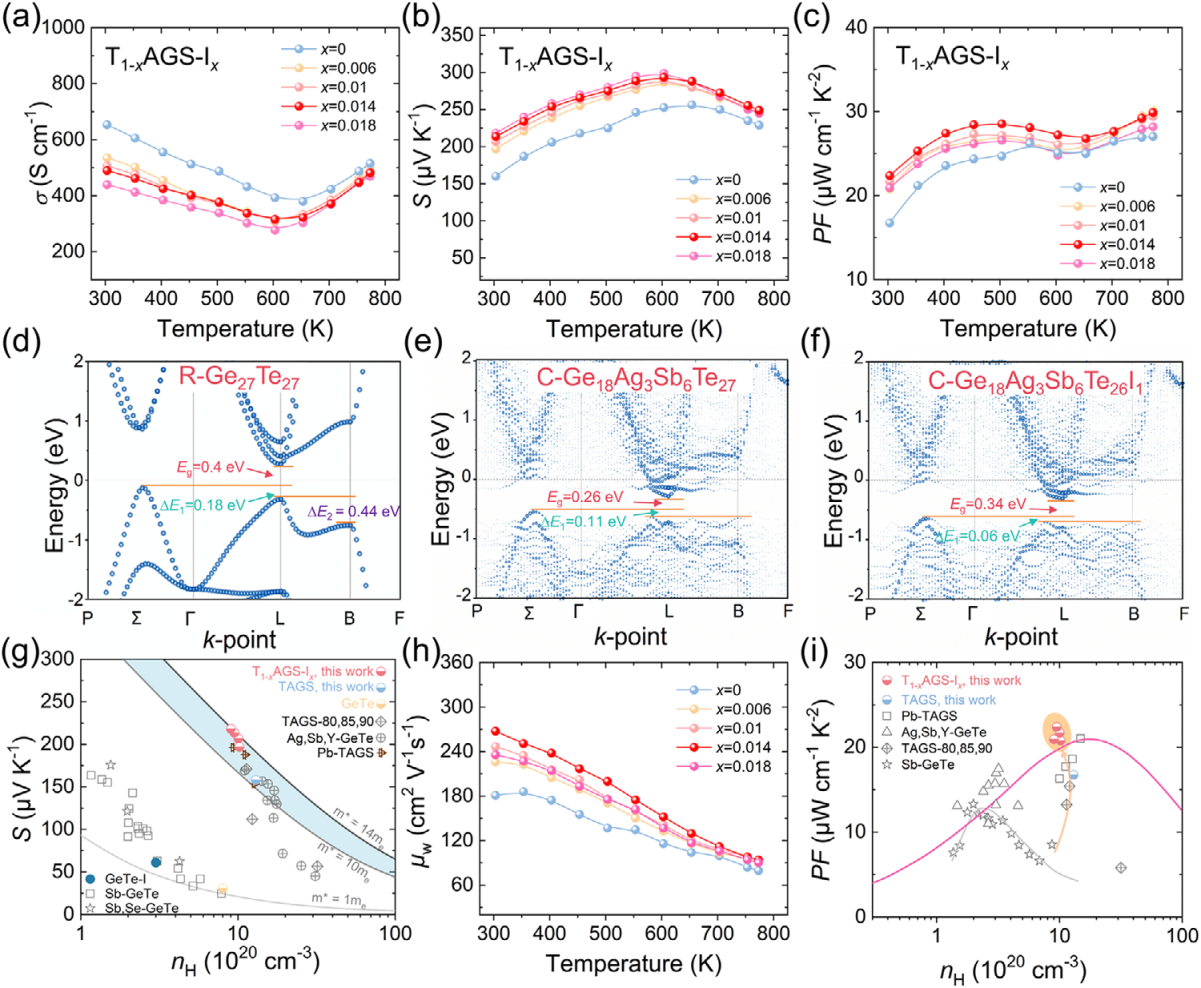
Electrical transport properties and electronic structure analysis of T_1‐_ *_x_* AGS‐I*_x_* (*x* = 0‐0.018) samples. (a) *σ* as a function of temperature, showing the influence of AgSbTe_2_ alloying and I doping on carrier transport. (b) *S* vs. temperature, highlighting the combined effect of band convergence and carrier concentration optimization. (c) Temperature‐dependent *PF*, demonstrating the enhanced electrical performance of I‐doped TAGS samples. (d‐f) Calculated electronic band structures of representative compositions: (d) rhombohedral Ge_27_Te_27_ (*R*‐Ge_27_Te_27_), (e) cubic Ge_18_Ag_3_Sb_6_Te_27_ (*C*‐Ge_18_Ag_3_Sb_6_Te_27_), and (f) I‐doped cubic Ge_18_Ag_3_Sb_6_Te_26_I_1_ (*C*‐Ge_18_Ag_3_Sb_6_Te_26_I_1_), showing band convergence and modification of the valence band maxima. (g) Pisarenko plot of *S* as a function of *n*_H_, with literature data [14, 41, 54, 55, 56]; solid lines represent theoretical single parabolic band (SPB) model predictions, illustrating the enhanced effective mass in T_1‐_ *_x_* AGS‐I*_x_* samples. (h) Weight mobility *µ*_w_ vs. temperature, calculated following the method of Snyder et al. [57], reflecting the synergistic optimization of carrier transport and band structure. (i) Comparison of *PF* of T_1‐_ *_x_* AGS‐I*_x_* samples with representative GeTe‐based materials from the literature [14, 41, 54, 56], demonstrating the superior performance achieved through combined alloying and I doping.

To clarify the origin of these electronic trends, Hall measurements were conducted (Figure S2). The substitution of I^−^ for Te^2−^ introduces additional electrons, thereby compensating native Ge vacancies and effectively reducing *n*_H_. Notably, *µ*_H_ remains largely unchanged across the series, indicating that the increased grain boundary density and local strain do not introduce detrimental carrier scattering centers. This preservation of *µ*_H_ remains is critical for maintaining a competitive *PF* and highlights the well‐managed defect landscape enabled by the coupled alloying‐halogen strategy. Importantly, GeTe is characterized by a two‐valence‐band structure, composed of a light valence band (LVB) and a heavy valence band (HVB). Therefore, *S* depends not only on *n*_H_ but also greatly on the DOS *m* ^*^. The large enhancement in *S* observed here cannot be solely attributed to the decrease in *n*_H_. Given that pristine GeTe exhibits a very low room‐temperature *S* of only 28.56 µV K^−1^, the remarkable increase to ∼218 µV K^−1^ indicates substantial band structure modification.

To elucidate the electronic origins underlying the enhanced electrical performance, DFT calculations were conducted on Ge_27_Te_27_, Ge_18_Ag_3_Sb_6_Te_27_, and Ge_18_Ag_3_Sb_6_Te_26_I_1_ supercells. The unfolded band structures, total DOS, and projected DOS (Figure 3d–f and Figures S2 and S3) clearly demonstrate that AgSbTe_2_ alloying and subsequent I doping induce substantial modifications to both the dispersive features near the Fermi level and the local electronic environment of Ge/Te. AgSbTe_2_ alloying stabilizes the cubic phase and triggers a pronounced convergence of the valence bands. In pristine Ge_27_Te_27_, the energy offset (*ΔE*) between LVB and HVB is ∼0.40 eV, with the *B*‐point VBM significantly lower than that at *L*. Upon alloying, *ΔE* rapidly decreases to 0.11 eV in Ge_18_Ag_3_Sb_6_Te_27_, yielding nearly degenerate *B*‐ and *L*‐point maxima. This convergence leads to a marked increase in DOS near the VBM, effectively enhancing the band degeneracy (*N_v_*) and the associated *S*. Simultaneously, the bandgap (*E*_g_) narrows from 0.40 to 0.26 eV, which may induce slight bipolar diffusion at elevated temperatures.

Strikingly, I doping further tunes the band structure by reinforcing the multivalley contribution without disrupting the cubic symmetry. The *ΔE* between these sub‐valence‐band maxima is reduced to only 0.06 eV in Ge_18_Ag_3_Sb_6_Te_26_I_1_, indicating a nearly complete VB convergence. Contrary to the typical halogen doping effects in rhombohedral GeTe, the present system already features a highly symmetric cubic lattice; therefore, the enhancement in *m* ^*^ originates from the increase in accessible valence‐band pockets rather than symmetry breaking or rhombohedral distortion. Notably, *E*_g_ widens to 0.34 eV upon I doping, which is directly responsible for the significantly improved *S* across the entire temperature range.

To further validate this band structure evolution, we constructed a Pisarenko plot using the single parabolic band (SPB) model under acoustic phonon‐dominated scattering. The experimental (*S*, *n*_H_) data of T_1‐_ *_x_* AGS‐I*_x_* (*x* = 0‐0.018) fall systematically above those of pristine GeTe‐based references (Figure 3g) [14, 41, 54, 55, 56]. The extracted room‐temperature *m* ^*^ increases from ∼10 *m*_e_ in the AgSbTe_2_‐alloyed sample to nearly 14 *m*_e_ after I doping, surpassing values previously reported for GeTe‐based solid solutions. These results provide strong evidence that the enhanced DOS *m* ^*^ is directly correlated with the enlarged *N_v_* induced by Ag/Sb/I co‐regulation.

The *µ*_w_, which reflects the intrinsic charge‐transport efficiency more explicitly, further corroborates this conclusion. I doping optimizes *n*_H_ while reshaping the electronic structure, yielding a substantial enhancement in *µ*_w_ (Figure 3h). This synergistic modulation leads to a remarkable increase in the *PF*. Compared with literature on similarly doped GeTe systems (Figure 3i) [14, 41, 54, 56], the T_1‐_ *_x_* AGS‐I*_x_* series exhibits simultaneously higher *PF* and suppressed bipolar broadening, enabling significantly improved high‐temperature *PF* values (Figure 3c). The optimized T_0.986_AGS‐I_0.014_ sample achieves an impressive average *PF*_ave_ of ∼27.3 µW·cm^−1^ K^−2^ from to 300–773 K, establishing a robust foundation for high‐power‐density TE device fabrication.

To clarify the structure‐property correlations governing the co‐optimization of charge and phonon transport and to confirm the formation of a true single‐phase solid solution in T_0.986_AGS‐I_0.014_, a comprehensive microstructural analysis was carried out using scanning electron microscopy (SEM), energy‐dispersive X‐ray spectroscopy (EDS), and multi‐scale transmission electron microscopy (TEM) characterizations. SEM‐EDS elemental mapping (Figure S5) demonstrates that all constituent elements exhibit a uniform spatial distribution in both TAGS and T_0.986_AGS‐I_0.014_, with no detectable elemental segregation or grain‐boundary enrichment except for an increase in the average atomic content of Ge. This observation is in full agreement with the XRD results.

Cross‐sectional SEM analysis (Figure 4a) reveals that the pristine TAGS matrix is fully dense with indistinct macroscopic grain boundaries, reflecting its inherently coarse‐grained microstructure. In contrast, the initial T_0.986_AGS‐I_0.014_ ingot exhibits a pronounced grain refinement (Figure 4b), as indicated by the yellow dotted lines. After ball milling and hot pressing, the microstructure further evolves toward a homogenized grain network, with grain sizes predominantly in the 2–3 µm range (Figure 4c and Figure S6). This refined grain structure forms a phonon scattering network, which is expected to significantly reduce *κ*_L_.

**FIGURE 4:**
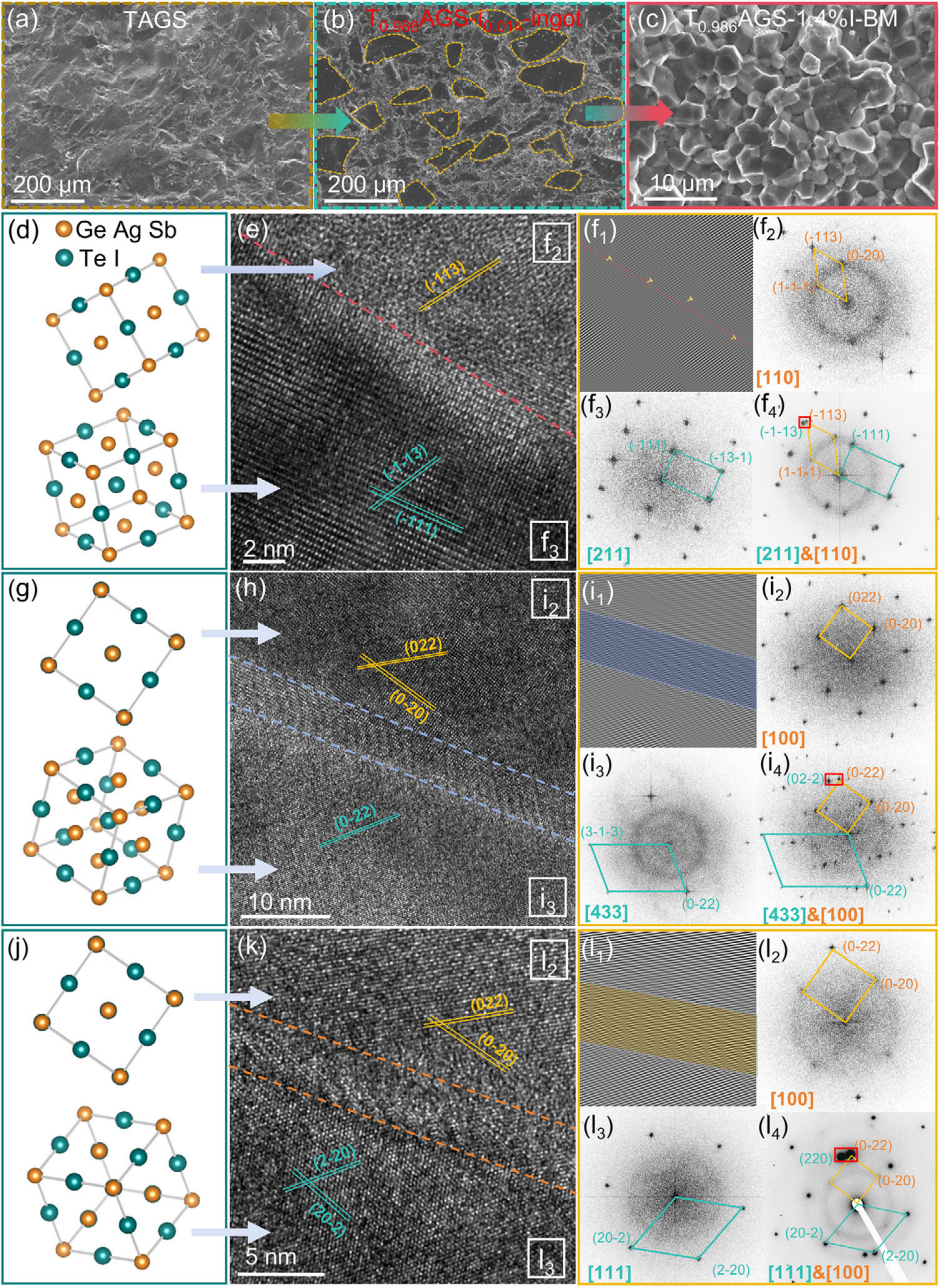
Microstructural characterization of the T_0.986_AGS‐I_0.014_ bulk specimen. Cross‐sectional microstructural analysis of (a) TAGS, (b) initial T_0.986_AGS‐I_0.014_ ingot, and (c) ball‐milled (BM) T_0.986_AGS‐I_0.014_ samples. (d) Atomic models of cubic‐GeTe along the [110] and [211] zone axes corresponding to the region highlighted in (e). (e) High‐resolution TEM (HRTEM) image of the marked region, showing adjacent grains with distinct orientations. (f_1_) Inverse FFT (IFFT) image emphasizing the semi‐coherent interface between grains. (f_2_,f_3_) FFT images of the individual grains corresponding to (e). (f_4_) FFT pattern of the entire region (e), highlighting slight lattice mismatch and coherent interface formation. (g) Atomic models of cubic‐GeTe along the [100] and [433] zone axes corresponding to the region in (h). (h) Enlarged HRTEM image illustrating grain‐boundary connectivity and interfacial structure. (i_1_) IFFT image emphasizing the interface matching zone between [100] and [433] grains. (i_2_,i_3_) FFT images of individual grains corresponding to (h). (i_4_) FFT pattern of the entire region in (h), confirming a semi‐coherent interface and lattice compatibility. (j) Atomic models of cubic‐GeTe along the [100] and [111] zone axes corresponding to the region in (k). (k) Enlarged HRTEM image highlighting intergranular structure and atomic alignment. (l_1_) IFFT image emphasizing the interface‐matched “buffer zone” between [100] and [111] grains. (l_2_,l_3_) FFT images of individual grains corresponding to (k). (l_4_) Selected‐area electron diffraction (SAED) pattern of the entire region in (k), confirming absence of secondary phases and high structural coherence at grain boundaries.

For heavily doped TAGS‐based semiconductors, the carrier mean free path (*l*_e_) is typically on the order of the lattice parameters [58, 59]. Therefore, grain size refinement has a negligible influence on carrier scattering. This expectation is fully supported by the short *l*_e_ extracted from transport measurements (Figure S7), confirming that the increased grain‐boundary density does not compromise *µ*_H_. Thus, microstructural engineering in this system predominantly modulates phonon rather than charge transport. To resolve the intergranular characteristics that ultimately determine *µ*_H_, high‐resolution TEM imaging and fast Fourier transform (FFT) analysis were conducted. Figure 4e and Figure S8 display two neighboring grains with distinct orientations, indexed as cubic GeTe along the [110] and [211] zone axes (Figure 4f_2_,f_3_). The corresponding atomic model is presented in Figure 4d. Despite their different orientations, the IFFT pattern (Figure 4f_4_) shows that the diffraction spots from specific planes of the two grains are nearly coincident, indicating closely matched *d*‐spacings and only a slight lattice mismatch (Figure 4f_1_). Such an interface is thus identified as semi‐coherent, which allows charge carriers to traverse grain boundaries with minimal additional resistance while still contributing to phonon scattering through interface strain and mismatch.

Further TEM characterizations reinforce this interfacial motif. Adjacent grains indexed along the [100] & [433] (Figure 4h,i and Figure S9) and [100] & [111] (Figure 4k,l and Figure S10) orientations show FFT patterns (Figure 4i_4_,l_4_), in which diffraction spots from specific planes of the two grains remain similar in *d*‐spacing but exhibit slightly enlarged spatial separation. The corresponding atomic models are presented in Figure 4g,j. This subtle offset suggests that atoms from adjacent grains extend toward each other across the interface to form an overlapping, interface‐matched transition region rather than a secondary phase (Figure 4i_1_,l_1_). These nanoscale “buffer zones” effectively accommodate the orientation mismatch, enabling coherent structural accommodation across grains. Such interface behavior—semi‐coherent boundaries with buffer‐like transition layers—is notably different from the high‐energy, high‐resistivity grain boundaries commonly observed in undoped or poorly matched GeTe‐based polycrystals. This distinct structural characteristic is attributed to the I‐doping‐induced fine‐tuning of the lattice constant, which enhances intergranular compatibility and suppresses the formation of electronically detrimental secondary phases. Therefore, the slight lattice mismatch observed therefore does not generate substantial interfacial potential barriers, ensuring that carrier transport remains continuous across randomly oriented grains.

As a result, the T_0.986_AGS‐I_0.014_ sample exhibits a unique microstructural configuration in which refined grains form a phonon scattering network, while semi‐coherent interfaces and interface‐matched transition regions maintain high‐quality electronic connectivity. This “electron‐transparent but phonon‐opaque” grain‐boundary network constitutes a critical structural advantage, enabling the simultaneous realization of robust *µ*_H_ and strong phonon scattering—both essential for achieving high TE performance.

Further analysis of randomly selected regions in the T_0.986_AGS‐I_0.014_ matrix reveals a rich variety of defect structures within the grains that strongly contribute to phonon scattering across multiple frequency regimes. As shown in Figure 5a, pronounced planar vacancy‐layer defects or van der Waals–like gaps (yellow arrows) are observed, replacing the coherent twin boundaries commonly present in conventional TAGS. Their existence is corroborated by the presence of orthogonal diffuse diffraction streaks along the defect‐extension direction in the corresponding FFT image (Figure 5b), demonstrating their long‐range planar continuity [60, 61]. At higher magnification, shorter and tortuous zigzag vacancy layers are also identified (Figure 5c). These fragmented vacancy planes arise mainly from the substitution of Ge/Te by larger Ag/Sb/I atoms, which impose local strain fields and exert a pinning effect on the migration of cation vacancies. This disrupts the formation of long‐range vacancy planes and leads to their tortuous fracture into shorter segments. Collectively, both extended and zigzag vacancy‐layer defects serve as effective mid‐frequency phonon scattering centers due to their geometry and spatial periodicity.

**FIGURE 5:**
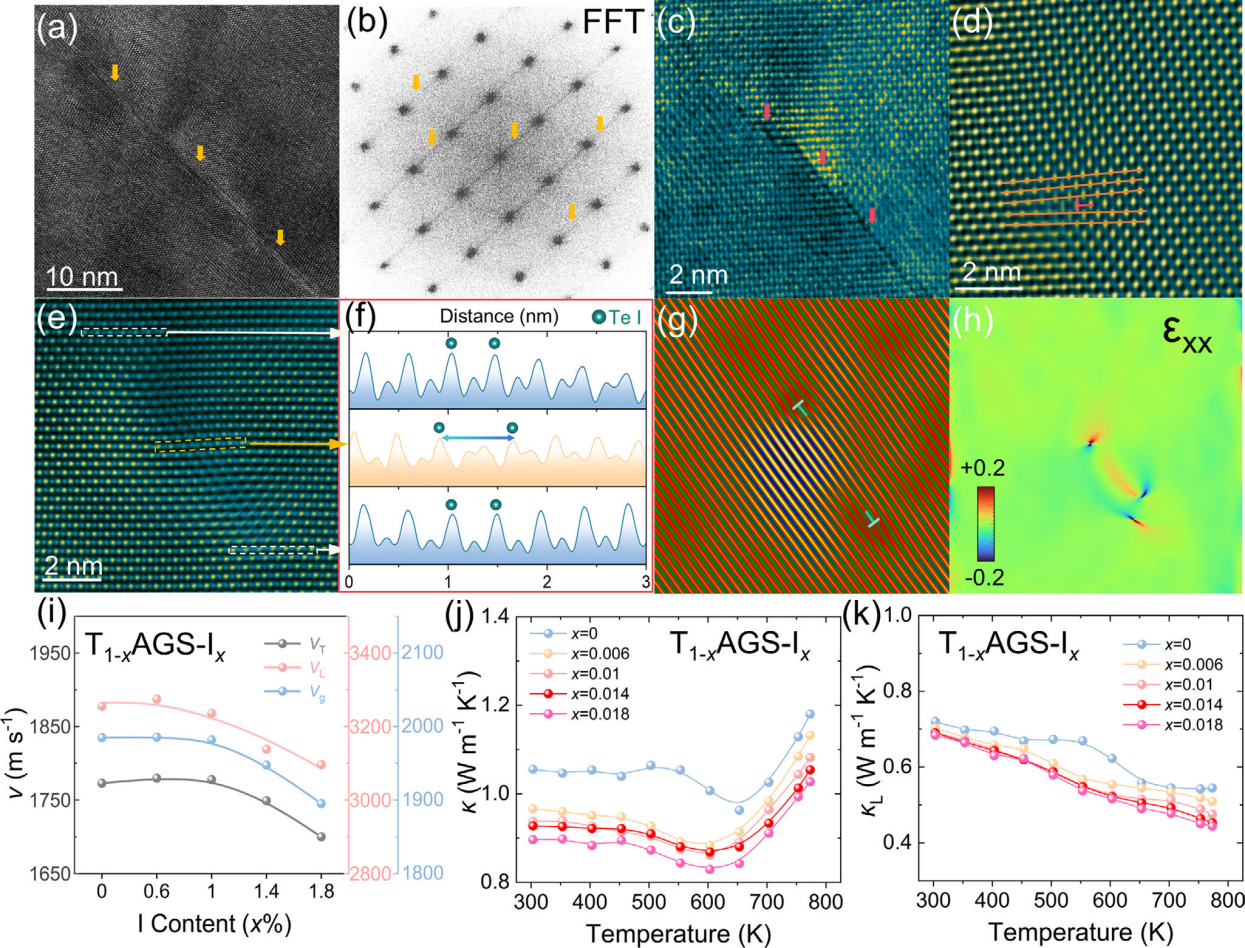
Thermal transport properties of T_0.986_AGS‐I_0.014_ and the underlying microstructural origins. (a) HRTEM image showing planar defect structures within the grains; (b) corresponding FFT pattern highlighting the periodicity of defect layers. (c) Zigzag vacancy layer structures with shorter segment lengths, illustrating the pinning effect of I and multi‐element alloying on cation vacancy migration. (d) HRTEM image of a representative dislocation within the grain. (e) Atomic‐scale irregularities in the lattice; (f) line intensity profiles across selected atomic sites revealing local atomic displacements. (g) IFFT image emphasizing local lattice distortions. (h) Geometric phase analysis (GPA) strain mapping of the dislocation region, indicating concentrated local strain that contributes to high‐frequency phonon scattering. (i) Room‐temperature sound velocity measurements, including average sound velocity (*v*_g_), transverse velocity (*v*_T_), and longitudinal velocity (*v*_L_), reflecting the effect of I doping on elastic properties and phonon propagation. (j) Temperature‐dependent total thermal conductivity (*κ*), showing significant reduction relative to pristine TAGS. (k) Lattice thermal conductivity (*κ*_L_), derived by subtracting the electronic contribution (*κ*_e_), highlighting the effectiveness of multi‐scale defect structures and grain refinement in suppressing phonon transport.

In addition to planar defects, pronounced dislocation networks are also observed within the grains (Figure 5d). The HRTEM image reveals bright Te atomic columns and comparatively weaker Ge columns (Figure 5e), consistent with the intrinsic cubic GeTe structure. However, detailed inspection of local atomic motifs and their corresponding intensity profiles (Figure 5f) uncovers irregular atomic displacements and expanded interatomic spacing. Such deviations are attributed to strong local fluctuations in the atomic mass and radius introduced by multi‐element alloying in the T_1‐_ *_x_* AGS‐I*_x_* system. These fluctuations distort the local bonding environment and promote the formation of dislocations (Figure 5g). Geometric phase analysis (GPA) further reveals pronounced strain localization around the dislocation cores (Figure 5h), indicating substantial local elastic distortions. These strained regions serve as potent scattering sites for high‐frequency phonons, thereby complementing the mid‐frequency scattering induced by planar vacancy layers and the low‐frequency scattering provided by grain boundaries.

The aforementioned multi‐scale microstructural reconstruction, characterized by grain refinement, the formation of high‐density dislocation arrays, and iodine‐induced point‐defect complexes, creates a hierarchical phonon‐scattering network that is highly favorable for suppressing *κ*_L_. Thus, I doping has a substantial impact on thermal phonon transport in TAGS‐based alloys. This conclusion is further validated by the sound velocity measurements shown in Figure 5i. With increasing I content, the average sound velocity (*v*_g_), transverse velocity (*v*_T_), and longitudinal velocity (*v*_L_) all exhibit a monotonic decrease compared with pristine TAGS, indicating a softened lattice and reduced phonon propagation capability. Such lattice softening is typically associated with enhanced mass and strain field fluctuation scattering, consistent with the structural disorder observed at both grain boundaries and intra‐grain regions.

Consequently, the T_0.986_AGS‐I_0.014_ sample exhibits a markedly reduced *κ* across the entire temperature range (Figure 5j) compared to the pristine TAGS. To elucidate the underlying mechanism, *κ*_e_ was extracted using the Wiedemann‐Franz law (*κ*_e_ = *Lσ*T). The Lorenz number (*L*) was estimated using the SPB model under the acoustic phonon scattering assumption, as detailed in the Supporting Information. As displayed in Figure S11, *κ*_e_ decreases with increasing I concentration. The slight increase in *κ*_e_ at elevated temperatures originates from the bipolar diffusion contribution, which follows the same temperature‐activated trend observed in *σ*. By subtracting *κ*_e_ from the measured *κ*, *κ*_L_ was obtained (Figure 5k). *κ*_L_ shows a pronounced decline upon I doping, confirming that phonon transport is predominantly impeded by the synergistic effects of (i) refined grain sizes that enhance boundary scattering, (ii) dense dislocation networks that introduce defect scattering, and (iii) I‐induced point defects that strongly scatter short‐wavelength phonons. Specifically, the T_0.986_AGS‐I_0.014_ sample alloy reaches an ultra‐low *κ*_L_ of ∼0.69 W m^−1^K^−1^ at 300 K and ∼0.45 W m^−1^ K^−1^ at 773 K—values approaching the amorphous limit of GeTe‐based systems. A comparison of *κ*_L_ with the state‐of‐the‐art GeTe‐ABX_2_ alloys reported in the literature is presented in Figure S12. The T_0.986_AGS‐I_0.014_ sample exhibits a significantly lower *κ*_L_ than most reported systems, underscoring the effectiveness of the engineered multi‐scale phonon scattering architecture. These results confirm that simultaneous control over grain‐boundary structure, intra‐grain defects, and point‐defect chemistry enables a highly competitive *κ*_L_, providing substantial benefits for thermal management and efficiency in high‐performance TE devices.

Ultimately, achieving high TE performance requires the simultaneous optimization of charge and phonon transport. As shown in Figure 6a, the representative T_0.986_AGS‐I_0.014_ sample exhibits markedly enhanced *µ*_w_/*κ*_L_ ratios of ∼3.87 × 10^4^ cm^3^ K J^−1^ V^−1^ at 300 K and ∼2.07 × 10^4^ cm^3^ K J^−1^ V^−1^ at 773 K, corresponding to 54% and 42% increases relative to pristine TAGS. Such improvement underscores the highly effective decoupling of electrons and phonons, wherein the strengthened *m* ^*^ and preserved *µ*_H_ coexist with the strongly scattered phonon system engineered by I doping and alloy‐defect interactions. Benefiting from (i) high *N_v_* introduced by Ag/Sb co‐alloying, (ii) suppressed rhombohedral distortion and stabilized cubic symmetry, and (iii) a hierarchical phonon scattering network dominated by grain boundaries, dislocations, and point‐defect complexes, all T_1‐_ *_x_* AGS‐I*_x_* alloys (*x* = 0‐0.018) show significantly elevated *zT* values across the full temperature range (Figure 6b). The clear correlation between *µ*_w_/*κ*_L_ and *zT* further demonstrates the coordinated optimization of charge and phonon conduits. The T_0.986_AGS‐I_0.014_ sample achieves a high *zT* of ∼0.7 at 300 K and a peak *zT* of ∼2.2 at 773 K, positioning it among the top‐performing cubic GeTe‐based thermoelectrics reported to date. Furthermore, good electrical repeatability and long‐term stability support its excellent thermal and structural stability under repeated heating‐cooling processes (Figure S13), confirming the reliability of the stabilized cubic phase.

**FIGURE 6:**
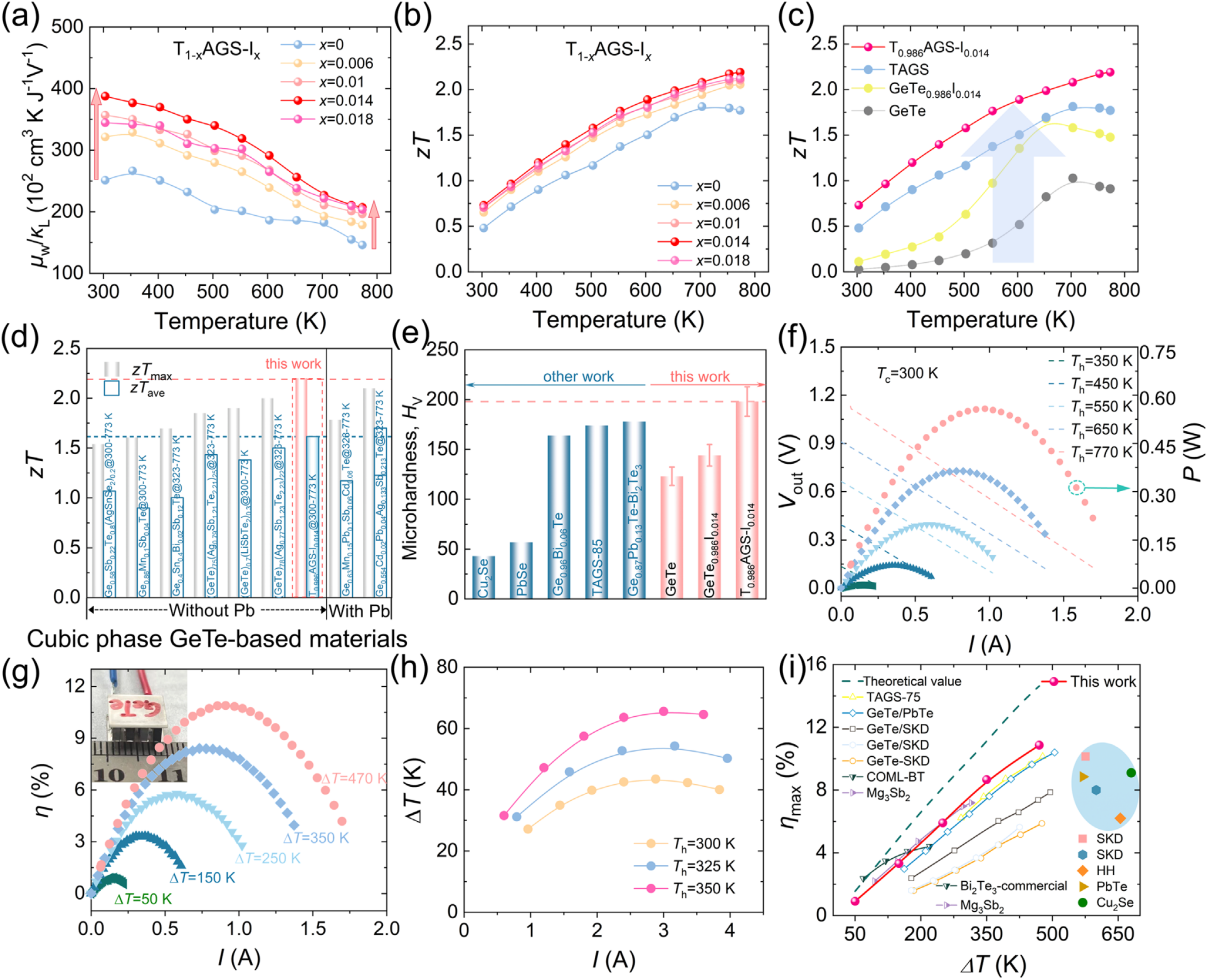
Thermoelectric performance, mechanical robustness, and device‐level evaluation of T_1‐_ *_x_* AGS‐I*_x_* samples. (a) Temperature‐dependent *µ*_w_/*κ*_L_ ratio, reflecting the synergistic optimization of charge and phonon transport. (b) Corresponding temperature‐dependent *zT* for T_1‐_ *_x_* AGS‐I*_x_* (*x* = 0‐0.018) samples, demonstrating the effect of I doping and grain refinement. (c) Comparison of *zT* for T_0.986_AGS‐I_0.014_ with GeTe, GeTe_0.986_I_0.014_, and pristine TAGS samples, highlighting the contribution of cubic‐phase stabilization and microstructural engineering. (d) Comparison of maximum *zT* (*zT*_max_) and average *zT* (*zT*_ave_) with representative state‐of‐the‐art cubic‐phase GeTe‐based thermoelectrics reported in the literature [34, 42, 44, 45, 46, 48, 62, 63]. (e) Vickers microhardness of T_1‐_ *_x_* AGS‐I*_x_* samples compared with reported thermoelectric materials [25, 62, 64, 65], demonstrating enhanced mechanical robustness from grain refinement and solid‐solution strengthening. (f) Output voltage (*V*) and power (*P*) of a 7‐pair *p*‐T_0.986_AGS‐I_0.014_/*n*‐PbTe device as a function of current (*I*) under various temperature differences (Δ*T*, *T*_c_ = 300 K). (g) Corresponding conversion efficiency (*η*) vs. current under varying Δ*T* for the same device, illustrating the influence of the high *zT*_ave_ of T_0.986_AGS‐I_0.014_ on device performance. (h) Thermoelectric cooling performance of a *p*‐T_0.986_AGS‐I_0.014_/*n*‐commercial Bi_2_Te_3_ device, showing maximum achievable Δ*T* under different *T*_h_. (i) Maximum conversion efficiency (*η*_max_) of the *p*‐GeTe/*n*‐PbTe thermoelectric device as a function of Δ*T*, compared with the literature‐reported devices [16, 34, 66, 67, 68, 69, 70, 71, 72, 73, 74, 75], confirming the competitive and dual‐mode (power generation and cooling) capabilities of the optimized I‐doped TAGS system.

To further delineate the contributions of cubic phase stabilization and grain refinement, a control sample of GeTe_0.986_I_0.014_ was synthesized and systematically evaluated (Figure S14). As presented in Figure 6c, although I doping alone improves the performance, the combined strategy of crystal symmetry engineering and mechanically induced grain refinement (via BM and alloying) results in substantially superior transport properties. This synergy enables an ultra‐high average *zT*_ave_ of ∼1.62, exceeding that of many recently reported Pb‐free GeTe derivatives and performing competitively even against state‐of‐the‐art Pb‐containing systems (Figure 6d). Beyond transport properties, the structural and mechanical robustness of T_0.986_AGS‐I_0.014_ plays a key role in enabling device‐level applicability. Grain refinement increases the barrier density against dislocation glide, while solid‐solution strengthening arising from Ag/Sb/I alloying enhances the lattice resistance to deformation and maintains cubic symmetry under load. These combined effects lead to significantly improved Vickers microhardness and compressive strength (Figures 6e and 1e). Such improvements at both micro‐ and macro‐scales are essential for practical device assembly, as they mitigate mechanical degradation during pressing, thermal cycling, and long‐term operation. Therefore, the exceptional mechanical reliability of the optimized composition offers an additional advantage beyond purely TE performance.

To further validate the practical applicability of the optimized T_0.986_AGS‐I_0.014_ composition, a 7‐pair TE device was assembled using our self‐developed *p*‐type legs and *n*‐type PbTe (Figure S15). The power‐generation capability was evaluated by measuring the single‐stage conversion efficiency. As shown in Figure 6f,g, and Figure S16a, the output voltage (*V*), output power (*P*), conversion efficiency (*η*), and heat flow (*Q*_c_) display the expected monotonic dependence on the operating current (*I*) under various Δ*T*. The device delivers a maximum *P* of 0.56 W, corresponding to a power density (*P*_d_ = *P*/*A*) of ∼2.03 W cm^−2^ (Figure S16b), which ranks competitively among mid‐temperature TE devices. The strong enhancement of *P*_d_ and *η* with increasing Δ*T* stems from the exceptionally high average *zT*_ave_ of T_0.986_AGS‐I_0.014_ across the mid‐to‐high temperature regime. Under an applied Δ*T* = 470 K with a cold‐side temperature (*T*_c_) of 300 K, the device achieves a maximum *η* of ∼11%, approaching the practical limit predicted for materials with comparable *zT*_ave_. This demonstrates that the synergistic optimization of charge transport, phonon scattering, and mechanical robustness directly translates into improved module‐level energy conversion.

In addition to power generation, the excellent room‐temperature *zT* (∼0.7) of T_0.986_AGS‐I_0.014_ suggests outstanding cooling capabilities. A 7‐pair cooling device fabricated using commercial *n*‐type Bi_2_Te_3_ (the corresponding thermoelectric transport properties are summarized in Table S2) exhibits a maximum Δ*T* of ∼43.5 K with a hot‐side temperature (*T*_h_) of 300 K, and up to ∼65.6 K at 350 K (Figure 6h and Figure S17). These performance metrics are on par with or exceed those of previously reported GeTe‐based coolers, further confirming the versatility of this cubic GeTe system for both cooling and power generation applications. A comparative efficiency analysis (Figure 6i) highlights that the T_0.986_AGS‐I_0.014_‐based device maintains a high *η* across the entire tested Δ*T* range and outperforms many reported Pb‐free GeTe devices. This advantage arises from (i) the stabilized cubic phase enabling suppressed bipolar conduction at elevated temperatures, (ii) the multi‐scale defect network that minimizes *κ*_L_, and (iii) the improved mechanical robustness that ensures reliable module assembly and sustained thermomechanical stability during operation.

## 3 Conclusion

In conclusion, we have developed a highly effective synergistic strategy to advance the performance ceiling of lead‐free GeTe thermoelectrics by simultaneously stabilizing the cubic phase and decoupling electron‐phonon transport. AgSbTe_2_ alloying suppresses the ferroelectric distortion and establishes a robust all‐cubic framework, while subsequent I doping induces refined single‐phase domains, multi‐degree‐of‐freedom grain‐boundary matching, and pronounced band degeneracy. These cooperative structural‐electronic optimizations substantially enhance the *µ*_w_/*κ*_L_ ratio, enabling an exceptional room‐temperature *zT* of ∼0.7 and a peak *zT* of ∼2.2 at 773 K. Meanwhile, the engineered multi‐scale defect architecture not only minimizes *κ*_L_ but also provides remarkable mechanical hardening, ensuring structural integrity during device fabrication and operation. These intrinsic material advantages are successfully translated into device‐level performance: a 7‐pair power‐generation device delivers a high *η* of ∼11%, and a cooling device assembled with commercial *n*‐type Bi_2_Te_3_ achieves sizeable Δ*T* of ∼43.5 K at 300 K and ∼65.6 K at 350 K, underscoring the dual‐mode application potential of the optimized cubic GeTe system. Overall, this study establishes a new paradigm for high‐performance Pb‐free thermoelectrics through lattice‐constant regulation, defect‐mode engineering, and intergranular matching. Beyond elevating the performance of GeTe, the presented design principles provide a generalizable platform for developing next‐generation TE materials and devices that unite high efficiency, mechanical robustness, and practical reliability across both power‐generation and solid‐state cooling applications.

## Author Contributions

**Xiaobo Tan**: Writing – original draft, investigation, validation, formal analysis, methodology. **Xuri Rao**: formal analysis, investigation. **Huangshui Ma**: formal analysis, data curation. **Jiaxing Luo**: formal analysis, investigation. **Fan Feng**: validation, formal analysis. **Zijian Lin**: investigation, validation. **Ruiheng Li**: validation, formal analysis. **Maoji Tian**: formal analysis, validation. **Siqi Huo**: validation, investigation. **Min Hong**: conceptualization, writing – review and editing, investigation, validation, formal analysis, software. **Ran Ang**: conceptualization, writing – review and editing, investigation, methodology, validation, formal analysis, resources, funding acquisition, project administration, supervision.

## Conflicts of Interest

The authors declare no conflict of interest.

## Supporting information




**Supporting File**: advs76668‐sup‐0001‐SuppMat.docx.

## Data Availability

The data that support the findings of this study are available from the corresponding author upon reasonable request.
