# Supplementary material for: Stabilized Cubic GeTe With Matched Grain‐Boundary Networks and Band Convergence for High‐Performance Dual‐Mode Thermoelectric Devices
Source: Adv Sci (Weinh). 2026 Jul 17:e76668. Online ahead of print. doi: 10.1002/advs.76668 (PMC13379259; doi:10.1002/advs.76668)
Supplement: Supplementary file 1 — Supporting File: advs76668‐sup‐0001‐SuppMat.docx. [file ADVS-9999-e76668-s001.docx]

**Supplementary materials**

**Stabilized Cubic GeTe with Matched Grain-Boundary Networks and Band Convergence for High-Performance Dual-Mode Thermoelectric Devices**

*Xiaobo Tan,^1^ Xuri Rao,^1^ Huangshui Ma, Jiaxing Luo, Fan Feng, Zijian Lin, Ruiheng Li, Maoji Tian, Siqi Huo,* *Min Hong*, Ran Ang**

X. Tan, X. Rao, J. Luo, F. Feng, Z. Lin, R. Li, M. Tian, R. Ang

Key Laboratory of Radiation Physics and Technology, Ministry of Education, Institute of Nuclear Science and Technology, Sichuan University, Chengdu 610064, China

Email: [rang@scu.edu.cn](mailto:rang@scu.edu.cn))

H. Ma, S. Huo, M. Hong

Centre for Future Materials, University of Southern Queensland, Springfield Campus, QLD 4300, Australia

Email: min.hong@unisq.edu.au

R. Ang

College of Physics, Sichuan University, Chengdu 610064, China

R. Ang

Institute of New Energy and Low-Carbon Technology, Sichuan University, Chengdu 610065, China

^1^(Xiaobo Tan and Xuri Rao contributed equally to this work)

Keywords: thermoelectrics, GeTe, cubic phase, grain boundary, cooling, power generation

1. **Experiment details**

**Sample synthesis:**

High-purity elements [Ge (99.999%), Te (99.999%), Ag (99.999%), TeI_4_ (99%), Sb (99.99%)] were weighed according to the nominal composition and sealed in evacuated quartz tubes under ~10^-5^ Torr. The mixtures were melted at 1173 Κ for 12 h to ensure complete homogenization and subsequently quenched rapidly in ice water to suppress unwanted phase segregation. The quenched ingots were then annealed at 773 Κ for 24 h to promote the formation of polycrystalline I-doped (GeTe)_78_(Ag_0.77_Sb_1.23_Te_2.23_)_22_ (T_1-_*_x_*AGS-I*_x_*, *x*= 0, 0.006, 0.01, 0.014, 0.018) solid solutions with a stable cubic phase, where x represents the atomic percentage (at%) of I substituting for Te. After annealing, the ingots were transferred into a glove box to maintain an inert atmosphere and weighed to achieve the desired mass ratios for T_1-_*_x_*AGS-I*_x_* compositions. The samples were then loaded into a stainless-steel jar and subjected to high-energy ball milling (MSK-SFM-3-I) at a vibration frequency of 600 rpm for 45 min to obtain fine powders with a uniform particle size and enhanced reactivity. The resulting powders were used for X-ray diffraction (XRD) characterization and consolidation via rapid hot pressing (HP). Hot pressing was carried out in an induction-heated graphite die at 773 Κ for 30 min under a uniaxial pressure of ~50 MPa. The final cylindrical specimens (diameter of ~12.5 mm) achieved a relative density of ≥98% of the theoretical value, as determined by the Archimedes method (**Table S1**), ensuring minimal porosity and optimal mechanical integrity for subsequent thermoelectric measurements.

**Structural characterization:**

Powder XRD patterns were collected at room temperature using Cu-Kα radiation (λ =1.5418 Å) on a high-resolution X-ray diffractometer to identify the phase composition and extract the lattice parameters. Rietveld refinement was performed to quantify the structural evolution and detect subtle lattice distortions. The microstructure, atomic-scale features, and chemical distribution of the samples were examined using a JEOL-NEOARM200F transmission electron microscope operated at 200 kV, equipped with a probe spherical-aberration corrector and an energy dispersive spectroscopy (EDS) detector. High-resolution TEM (HRTEM), selected-area electron diffraction (SAED), fast Fourier transform (FFT) analyses, and high-angle annular dark-field scanning TEM (HAADF-STEM) imaging were employed to resolve the grain-boundary structures, defect features, and crystallographic orientation relationships within the multiphase domains.

**Transport property measurements:**

The electrical conductivity (*σ*) and Seebeck coefficient (*S*) were simultaneously measured using a CTApro measurement system (Beijing Cryoall Science and Technology Co., Ltd. China) under a low-pressure He atmosphere to minimize convective heat loss. The total thermal conductivity (*κ*) was obtained from *κ*_total_ = *ρC*_p_*D*, where *ρ* is the bulk density (determined by the Archimedes method), *C*_p_ is the specific heat capacity, and *D* is the thermal diffusivity. The *D* was determined via the laser flash method using a Netzsch LFA 467 instrument (Netzsch, Shanghai, China). Hall measurements were performed using the Van der Pauw technique under a magnetic field of 1.5 *T*. The Hall carrier concentration (*n*_H_) and mobility (*μ*) were derived from *n*_H_ = 1/(*eR*_H_) and *μ* = *σR*_H_, respectively, where *R*_H_ is the Hall coefficient and *e* is the electron charge. Sound velocities (*v*), including the longitudinal (*v*_L_) and transverse (*v*_T_) modes, were measured using the pulse-echo method with an Olympus-NDT pulser/receiver and a Keysight oscilloscope. The average sound velocity (*v*_g_) was calculated using standard elastic wave relations. The mechanical properties were assessed at both the micro- and macro-scales.

**Mechanical properties:**

Vickers microhardness (*H*_v_) was measured using an HVS-2000 tester with a load of 1 N and a dwell time of 10 s. Compressive strain-stress curves were recorded on a universal testing machine at a loading rate of 0.02 mm/min to evaluate bulk mechanical resilience and deformation behavior.

**XANES spectroscopy measurements:**

The X-ray absorption near-edge structure (XANES) spectroscopy experiments were carried out at the BL14W1 beamline (18 KeV) in Shanghai Synchrotron Radiation Facility (SSRF). We measured the XAFS of Ge *K*-edge for GeTe and T_0.986_AGS-I_0.014_ in fluorescence mode.

**Density functional theory (DFT) calculations:**

First-principles calculations were performed within the framework of DFT using the Vienna Ab-initio Simulation Package (VASP).^[1-3]^ Structural optimization and electronic structure calculations were complemented by the VASPKIT package for post-processing.^[4]^ The electron exchange-correlation interaction was treated using the Perdew-Burke-Ernzerhof (PBE) functional within the generalized gradient approximation (GGA).^[5]^ A plane-wave cutoff energy of 450 eV was adopted to ensure sufficient accuracy in the total-energy convergence. A 3×3×3 supercell (Ge_27_Te_27_) constructed from a GeTe primitive cell was employed to calculate the formation energies, defect energetics, and electronic band structures. The Brillouin zone was sampled using *Γ*-centered *k*-point meshes appropriately scaled according to the supercell size. All structures were fully relaxed until the residual forces were below 0.01 eV Å^-1^. Spin-orbit coupling was not included due to the large supercell size and computational cost. Given that the present study focuses on relative electronic-structure trends rather than absolute band gaps, this approximation is not expected to affect the main conclusions. The supercell structures were constructed by randomly substituting Ag and Sb on Ge sites and I on Te sites, while maintaining overall stoichiometry and reasonable local coordination environments.

**Thermoelectric device fabrication and performance evaluation:**

A 7-pair thermoelectric device was assembled using *p*-type T_0.986_AGS-I_0.014_ and *n*-type PbTe legs, each with dimensions of 1.4 mm×1.4 mm×3.0 mm. To prevent interfacial interdiffusion during high-temperature operation, both the *p*- and *n*-type legs were electroplated with a Ni diffusion-barrier layer. For device fabrication, the thermoelectric legs were directly bonded in series onto a copper direct-bonded copper (DBC) ceramic substrate using Ag-based conductive solder. The output power and conversion efficiency were measured using a custom-built test system that was calibrated and validated using a Mini-PEM thermal measurement system (Advance Riko, Japan). All measurements were conducted under vacuum to eliminate convective heat loss. A 7-pair cooling device was similarly fabricated by bonding *p*-type T_0.986_AGS-I_0.014_ with commercial *n*-type Bi_2_Te_3_ (the thermoelectric transport properties are summarized in **Table S2**) legs using Sn-based solder on a DBC substrate. The maximum cooling temperature difference was determined as Δ*T*_max_=*T*_h_ – *T*_c_. All measurements were performed in vacuum using a home-made measurement system. The measurement protocol and underlying principles follow well-established methodologies reported in Refs.^[6-8]^

1. **Modeling based on the single parabolic band (SPB) framework**

To quantitatively evaluate the electronic transport behavior of GeTe-based alloys, the classical SPB model was employed under the assumption that carrier scattering is dominated by acoustic phonons. Within this framework, the Seebeck coefficient *S* is expressed as:

$S=\frac{k_{b}}{e}[\frac{\left( r+5/2 \right)F_{r+3/2}\left( \eta\right)}{\left( r+3/2 \right)F_{r+1/2}\left( \eta\right)}-\eta]$ (S1)

where *η* is the reduced chemical potential, *k*_B_ is the Boltzmann constant, *e* is the electron charge, *r* is the scattering factor, and *F*_r_(*η*) is the Fermi integral of order *r*.

The Hall carrier concentration $n_{H}$ is expressed as:

$n_{H}=4\pi{[\frac{{2m^{*}k}_{B}T}{h^{2}}]}^{3/2} F_{1/2}$ (S2)

where *m*^*^ is the density-of-states effective mass (accounting for band degeneracy), *h* is the Plank’s constant, and *T* is the absolute temperature.

The mobility *μ_H_* is expressed as:

$\mu_{H}=\mu_{0}\frac{F_{-1/2}}{{2F}_{0}}=\frac{\tau_{0}e}{m^{*}}\frac{F_{-1/2}}{{2F}_{0}}$ (S3)

where $\tau_{0}$ is the relaxation time, which is closely related to the energy in the case of acoustic phonon scattering: $\tau_{0}=\frac{h^{4}C_{l}}{8\sqrt{2}\pi^{3}{E_{def}}^{2}{m^{*}kT}^{3/2}}$, where *C*_l_ is a parameter determined by the combination of the elastic constant, and *E_def_* is a combination of deformation potentials for multivalley systems.

The Hall factor *A* is written as:

$A=\frac{3}{2}F_{1/2}\left( \eta\right)\frac{F_{{-1}/2}}{2F_{0}^{2}}$ (S4)

The Hall factor captures both the dominant scattering mechanism and possible band anisotropy. In the SPB framework used here, band anisotropy is neglected, allowing *A* to depend only on the scattering physics characterized by *r*.

The Lorenz number *L*, required for calculating the lattice thermal conductivity, is determined by:

$L=\frac{\kappa_{B}^{2}}{e^{2}}\frac{3F_{0}F_{2}-4F_{1}^{2}}{F_{0}^{2}}$ (S5)

In the equations above, the integral $F_{j}$ is defined by:

$F_{j}\left( \eta\right)=\int_{0}^{\infty} \frac{\xi^{j}d\xi}{1+e^{(\xi-\eta)}}$ (S6)


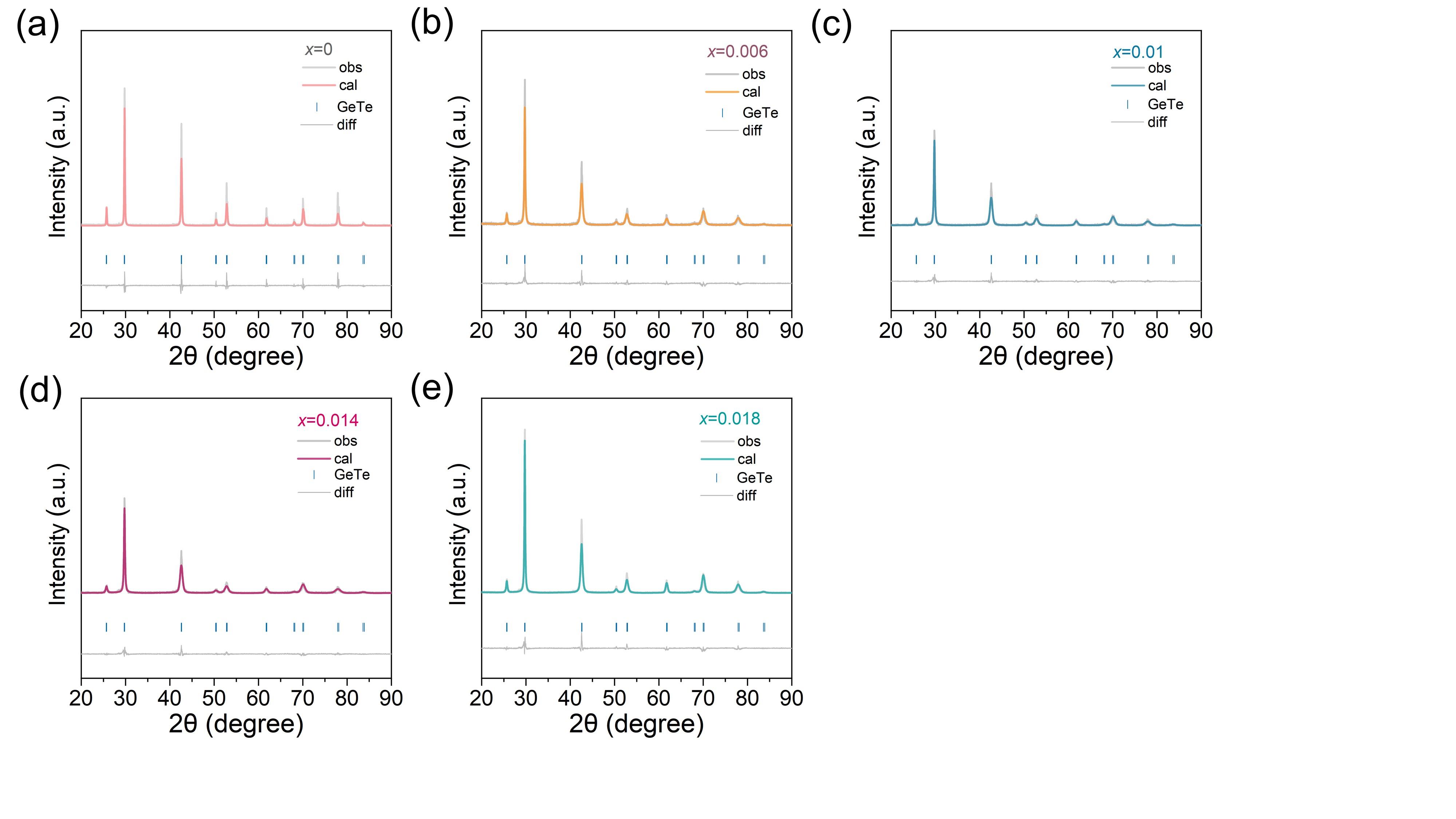


**Figure S1**. Rietveld refinement results for T_1-_*_x_*AGS-I*_x_* (*x* = 0-0.018) samples with (a) *x*=0, (b) *x*=0.006, (c) *x*=0.010, (d) *x*=0.014, and (e) *x*=0.018.


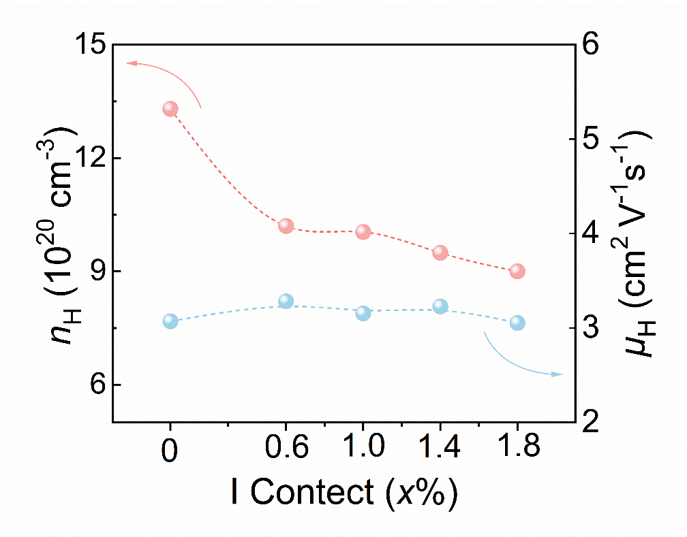


**Figure S2**. Room-temperature Hall Carrier concentration *n*_H_ and carrier mobility *μ*_H_ of the T_1-_*_x_*AGS-I*_x_* (*x*= 0-0.018) samples.


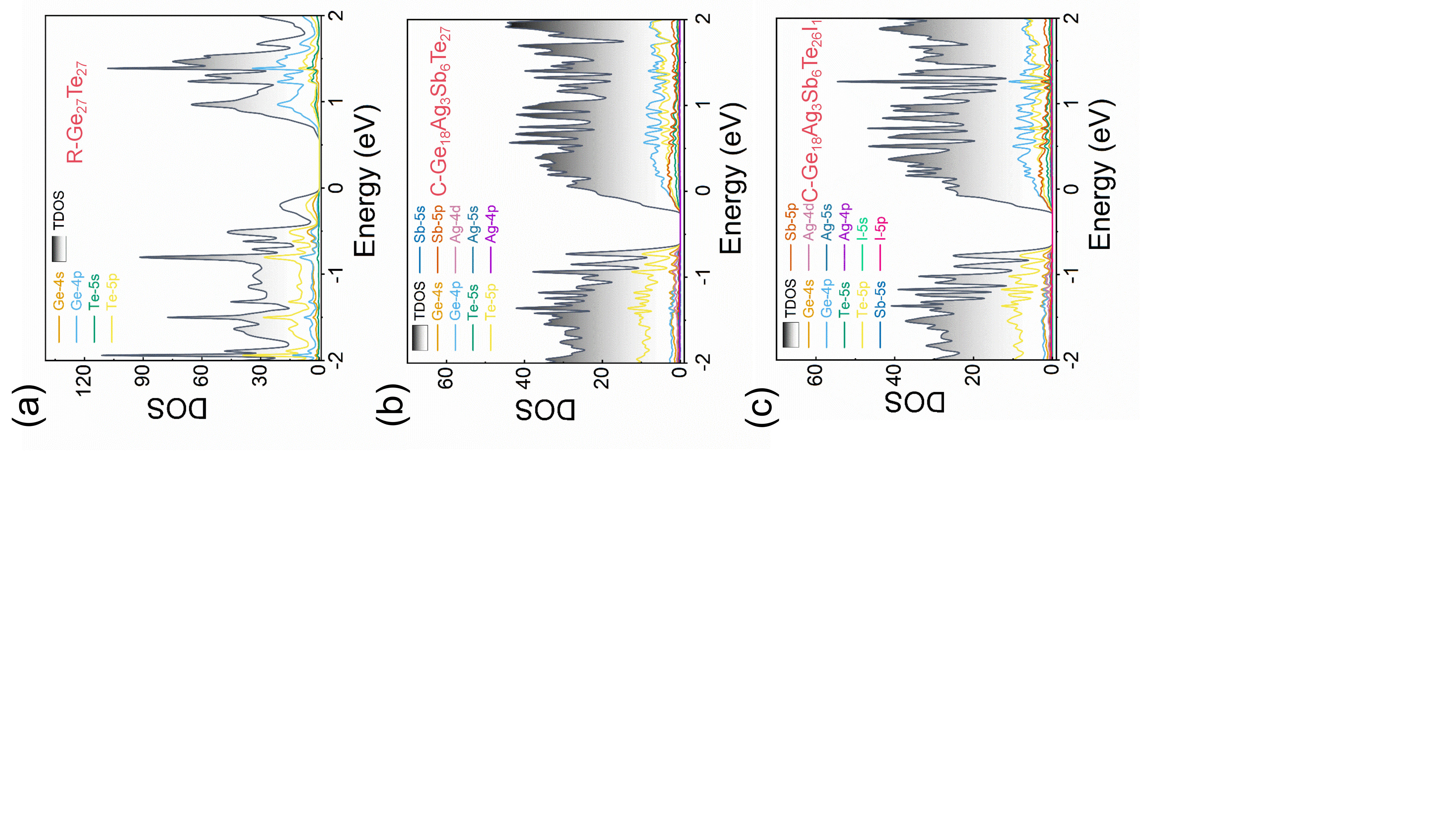


**Figure S3**. Calculated total density of states (TDOS) and projected DOS for (a) rhombohedral Ge_27_Te_27_ (*R*-Ge_27_Te_27_), (b) cubic Ag-Sb alloyed Ge_18_Ag_3_Sb_6_Te_27_ (*C*-Ge_18_Ag_3_Sb_6_Te_27_), and (c) I-doped cubic (*C*-Ge_18_Ag_3_Sb_6_Te_26_I_1_).


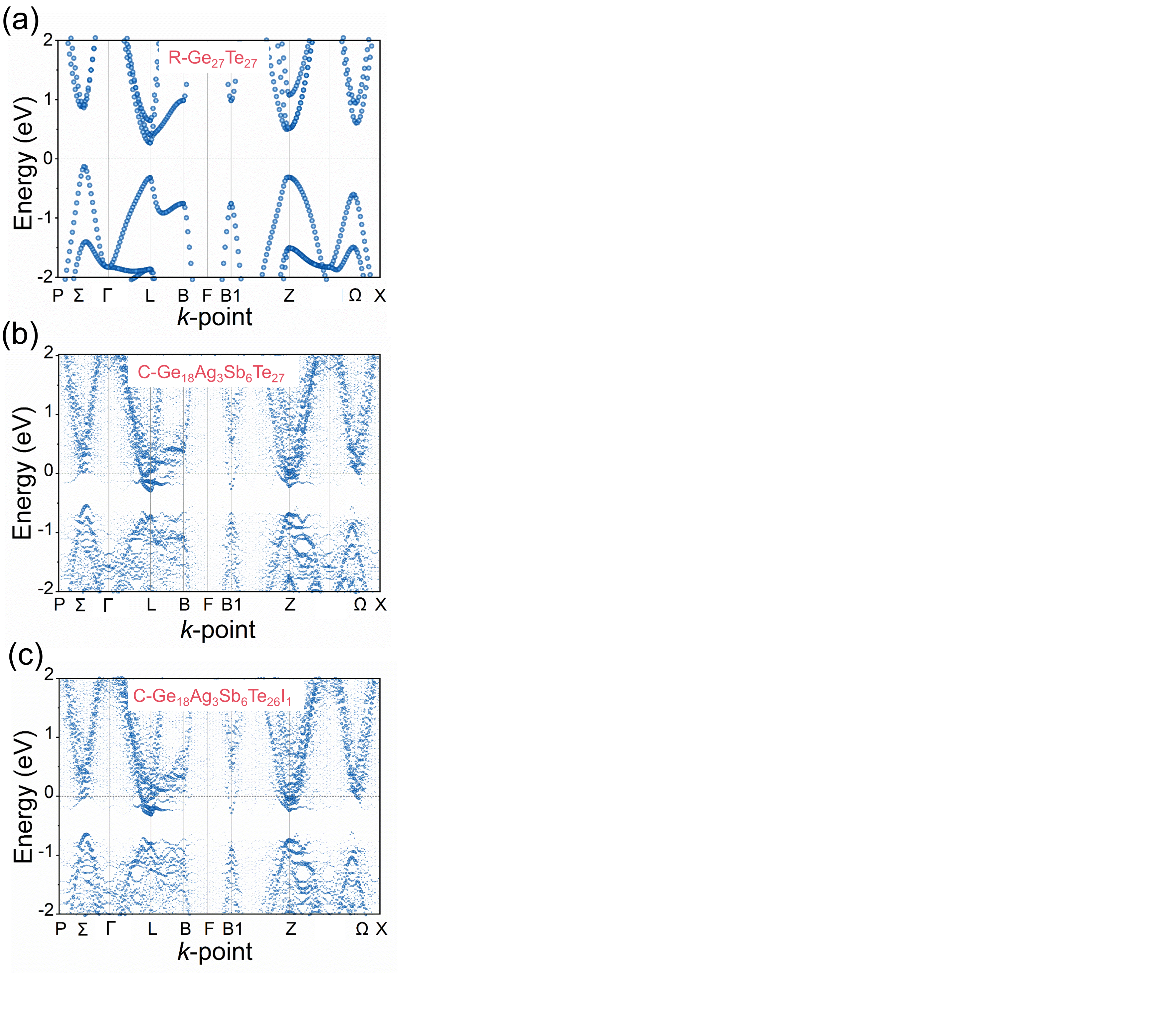


**Figure S4**. Calculated electronic band structures of (a) *R*-Ge_27_Te_27_, (b) *C*-Ge_18_Ag_3_Sb_6_Te_27_, and (c) *C*-Ge_18_Ag_3_Sb_6_Te_26_I_1_.


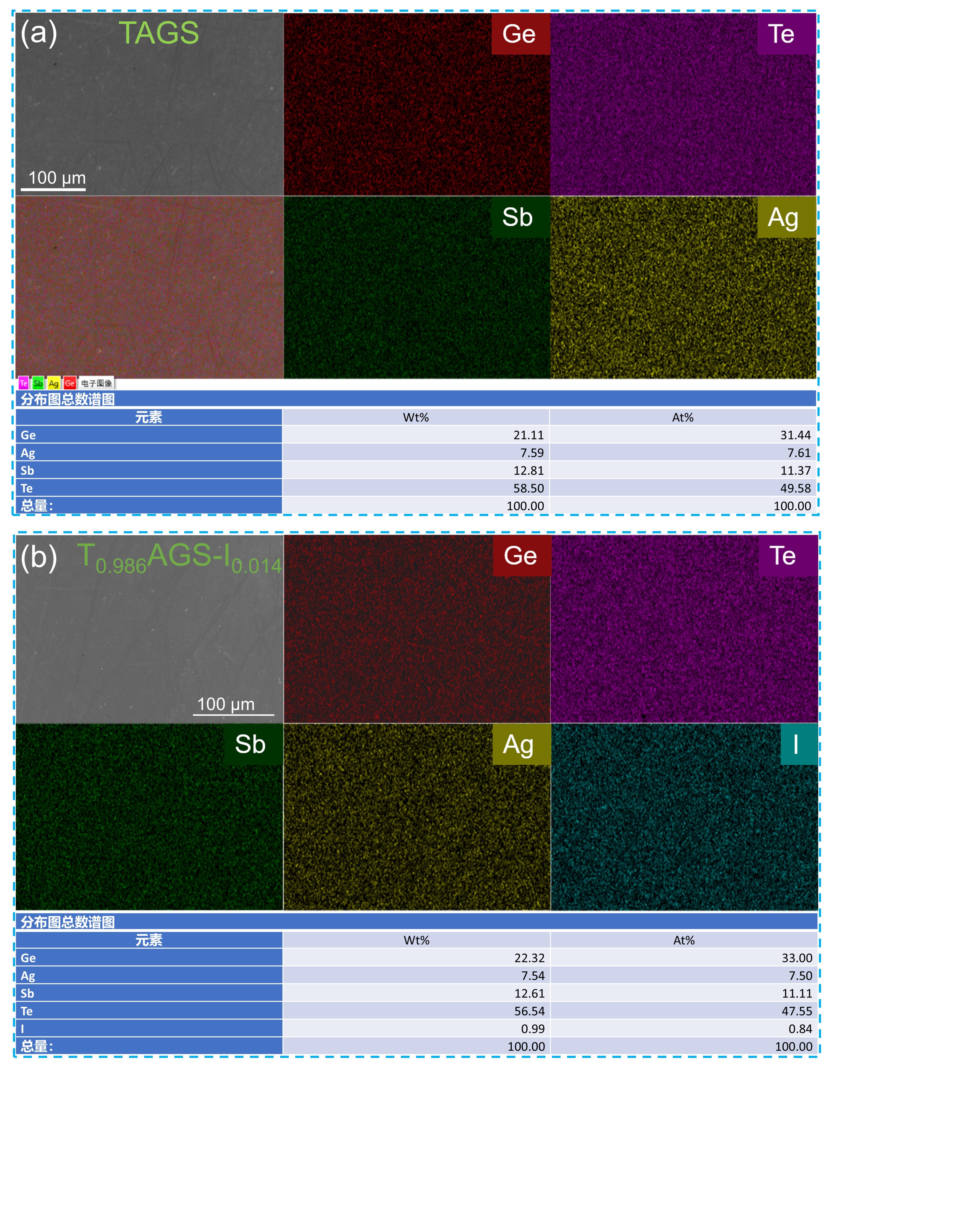


**Figure S5**. SEM images and corresponding EDS elemental mappings of Ge, Te, Sb, Ag, and I in the (a) TAGS and (b) ball-milled (BM) T_0.986_AGS-I_0.01_ samples.


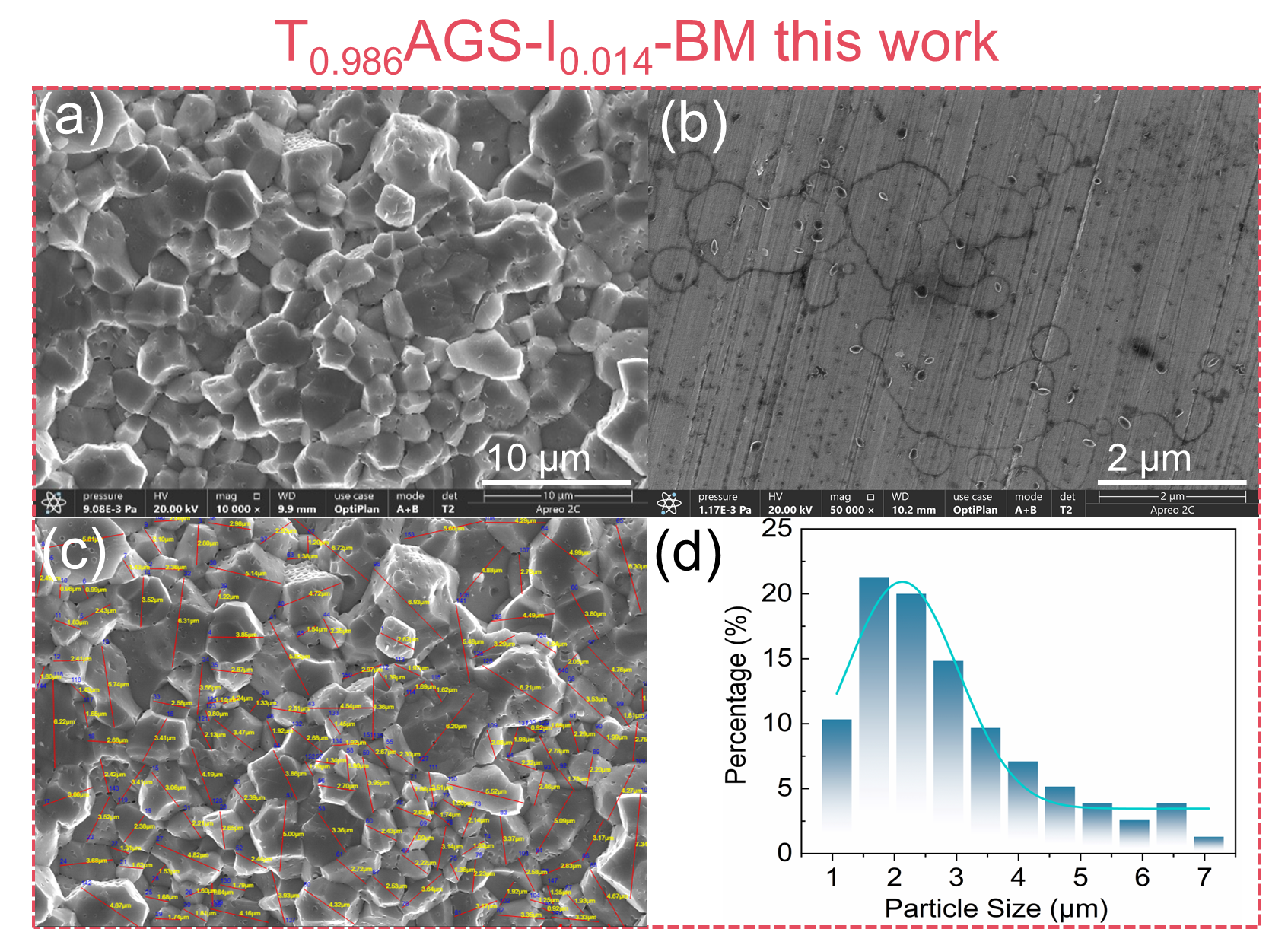


**Figure S6**. Cross-sectional microstructural analysis of (a) BM T_0.986_AGS-I_0.014_ samples. (b) Back-scattered electron microscopy image of the BM T_0.986_AGS-I_0.014_ sample. (c-d) Grain size distribution statistics derived from cross-sectional analysis, indicating effective grain refinement after ball milling and I doping.





**Figure S7**. Carrier mean free path (*l*_e_) of the T_1-_*_x_*AGS-I*_x_* (*x* = 0-0.018) samples as a function of I content.


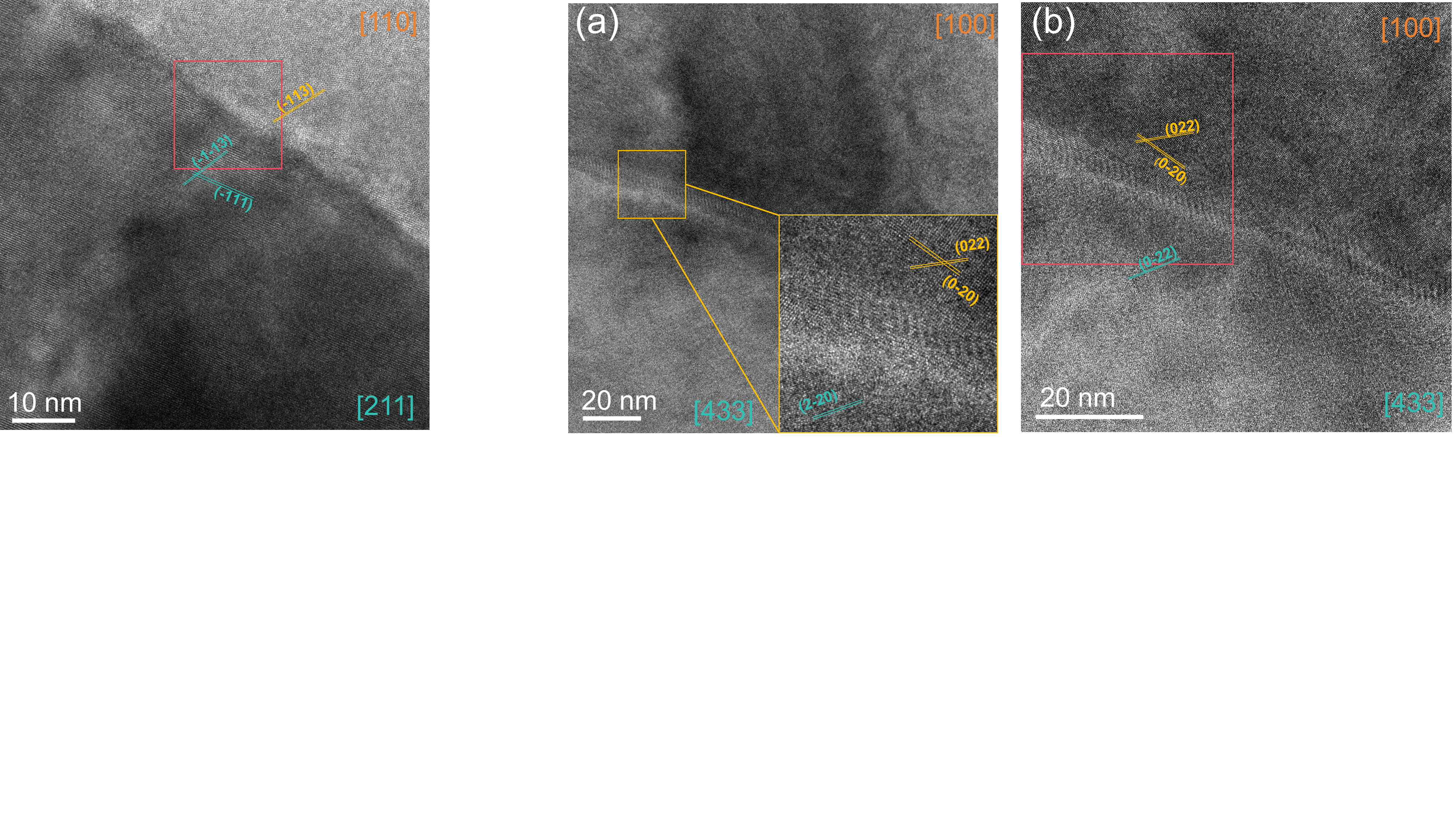


**Figure S8**. High-resolution TEM images of the T_0.986_AGS-I_0.014_ specimen were acquired along the [110] and [211] zone axes. The red-marked region corresponds to the area analyzed in Figure 4e of the main manuscript.


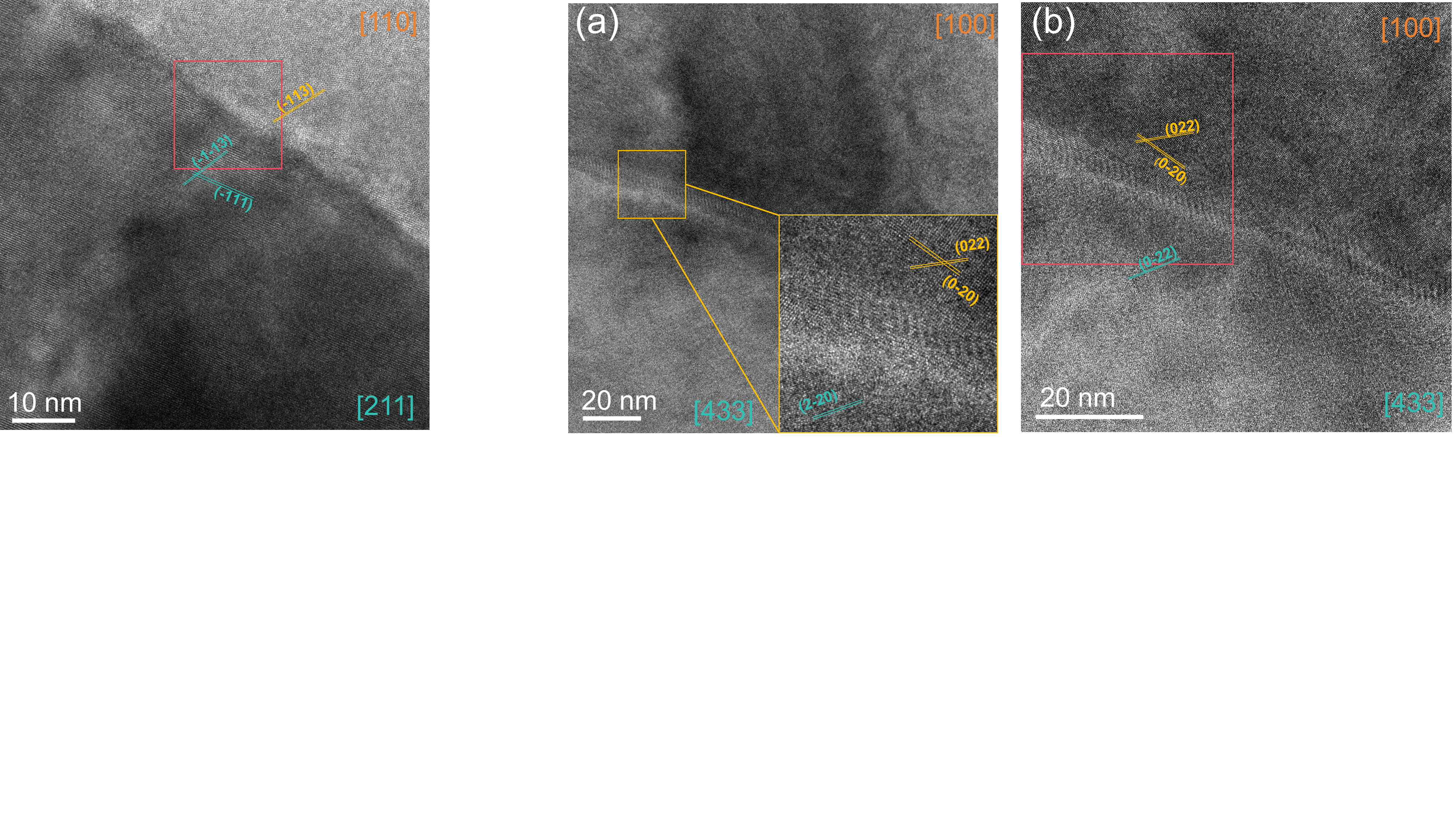


**Figure S9**. High-resolution TEM images of the T_0.986_AGS-I_0.014_ sample: (a) region along the [100] and [433] zone axes and (b) enlarged view of the interface. The red-marked area in (b) corresponds to the region highlighted in Figure 4h of the main manuscript.


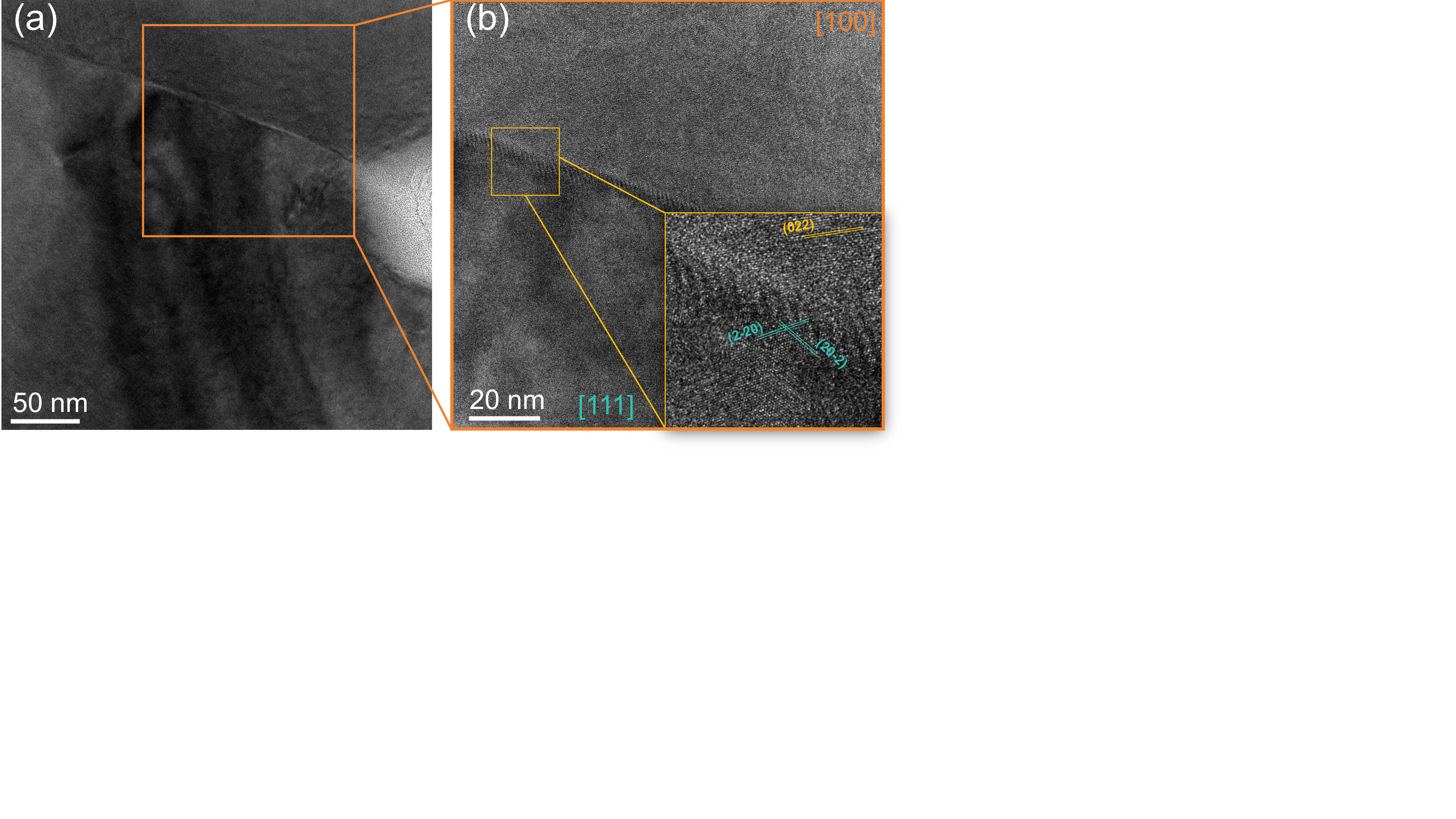


**Figure S10**. High-resolution TEM images of the T_0.986_AGS-I_0.014_ sample: (a) region along the [100] and [111] zone axes and (b) magnified view of the area highlighted in (a).


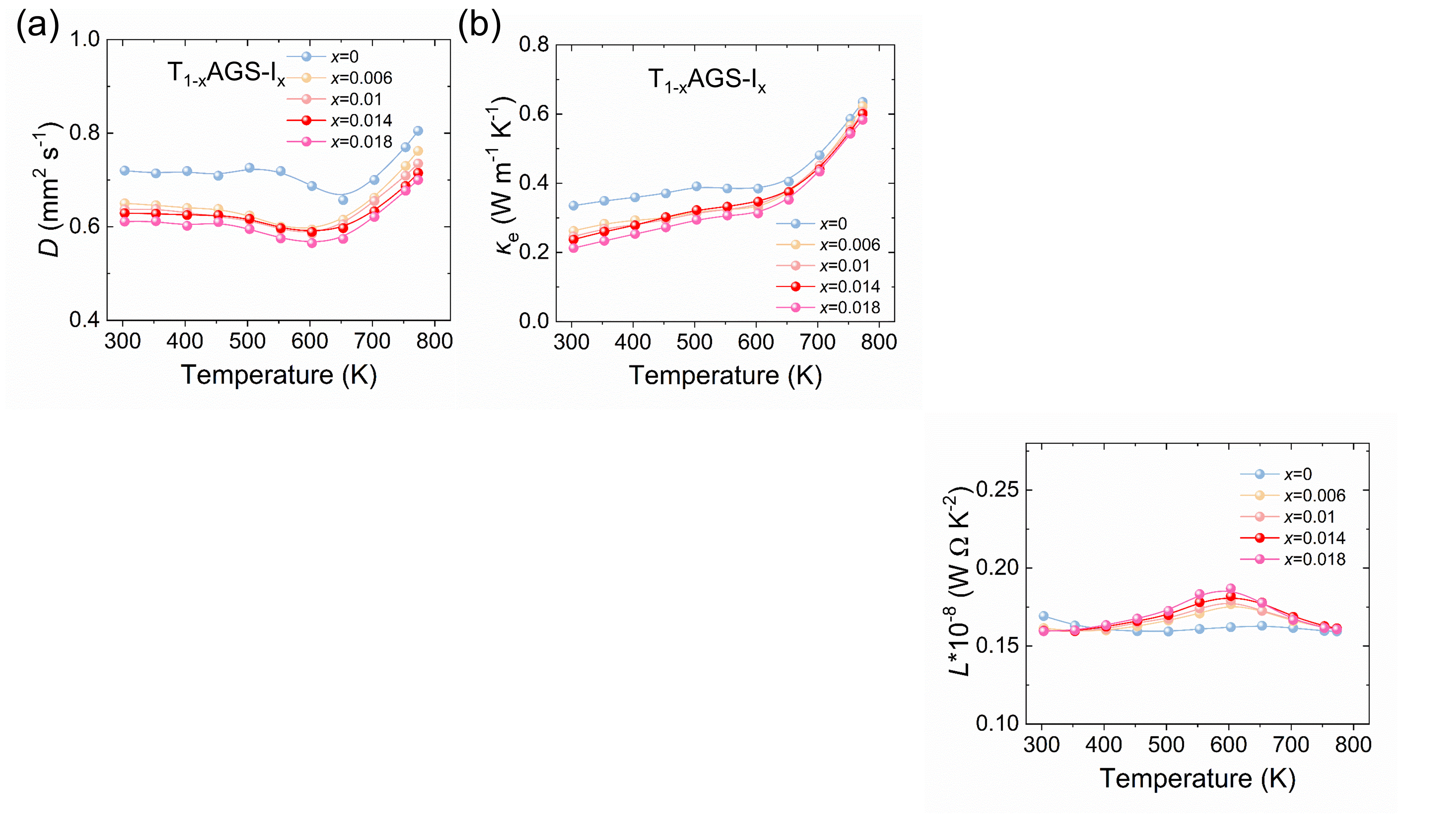


**Figure S11**. Temperature-dependent (a) thermal diffusivity (*D*) and (b) electronic thermal conductivity (*κ*_e_) of the T_1-_*_x_*AGS-I*_x_* (*x* = 0-0.018) samples.


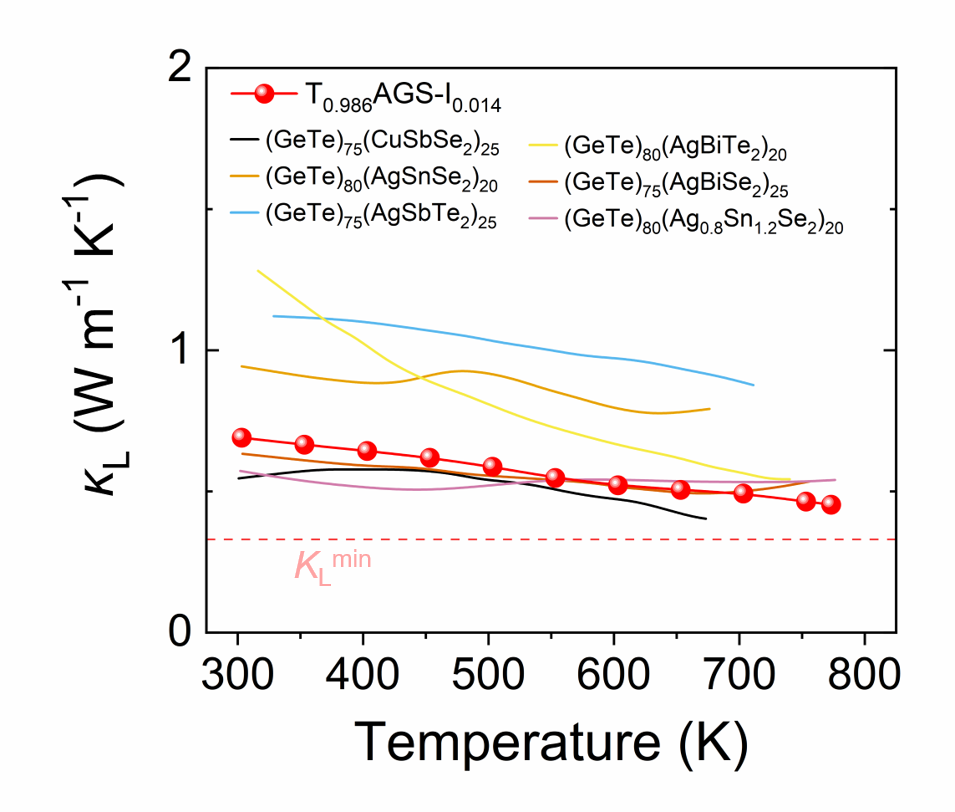


**Figure S12**. Comparative analysis of lattice thermal conductivity *κ*_L_ of the T_0.986_AGS-I_0.014_ sample against representative GeTe-ABX₂ alloy systems reported in the literature.^[9-12]^


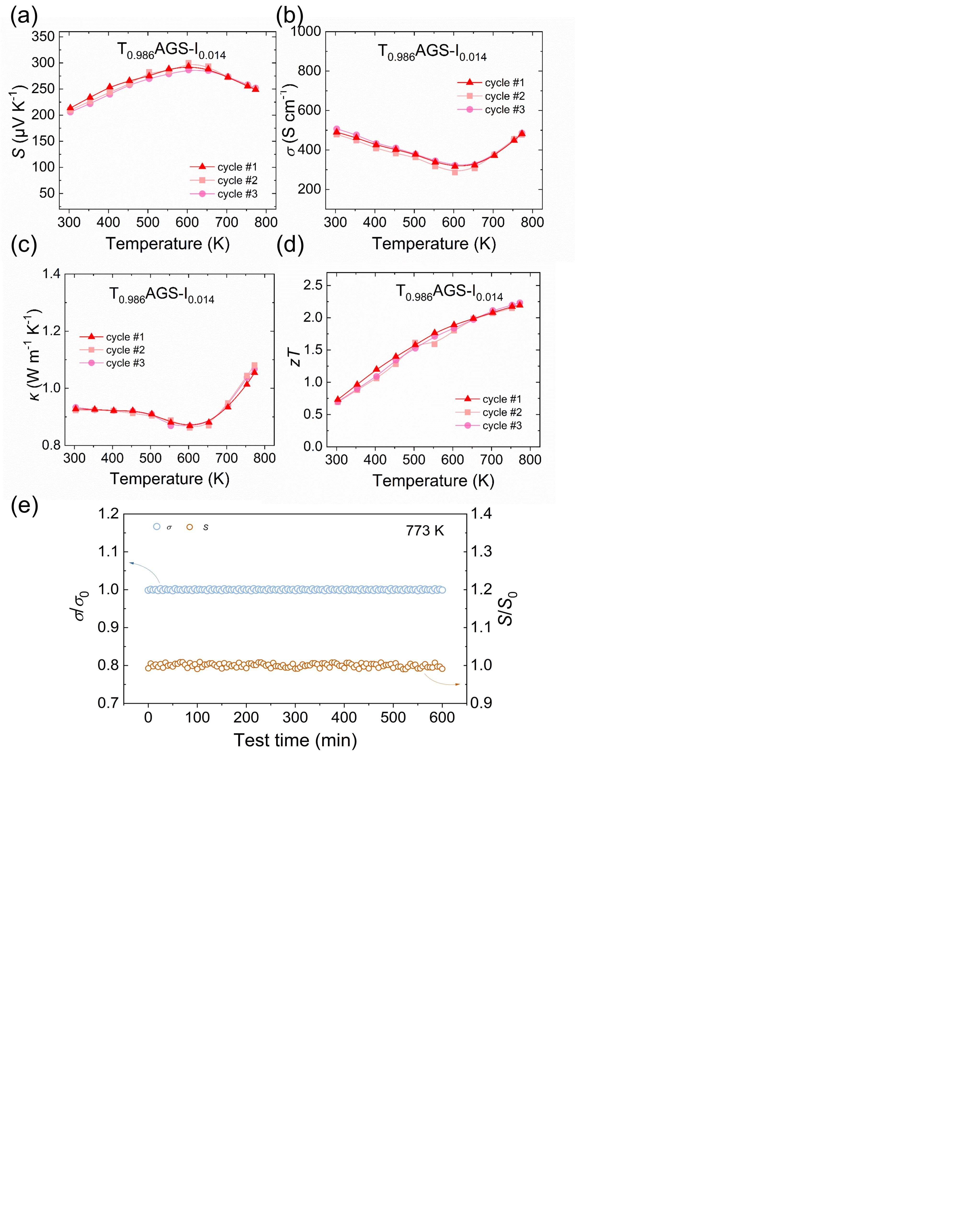


**Figure S13**. Repeatability and thermal stability of thermoelectric transport properties of T_0.986_AGS-I_0.014_. Temperature-dependent measurements of (a) Seebeck coefficient *S*, (b) electrical conductivity *σ*, (c) total thermal conductivity *κ*, and (d) figure-of-merit *zT*. (e)Stability of the electrical properties in T_0.986_AGS-I_0.014._


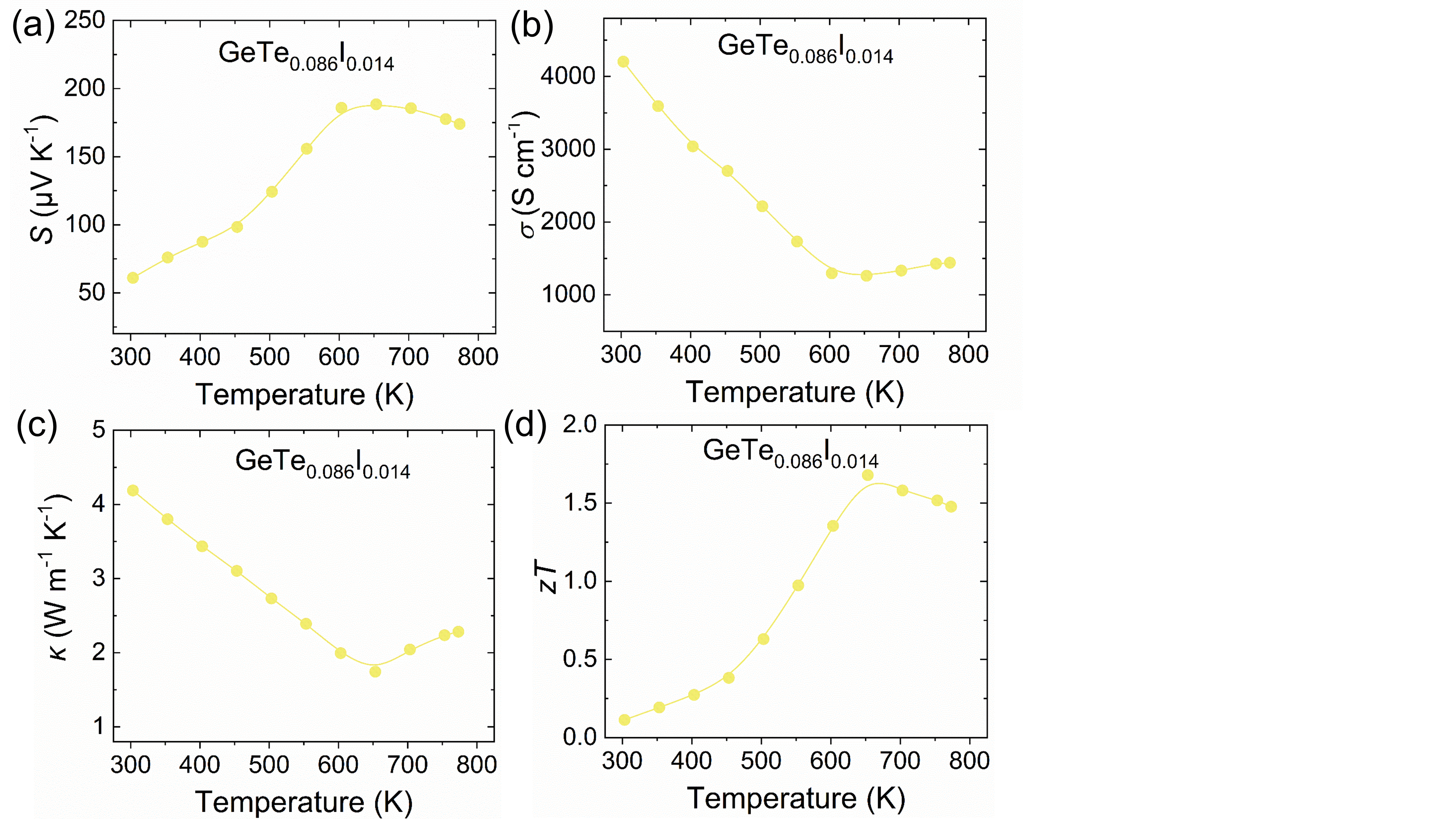


**Figure S14**. Thermoelectric transport properties of the GeTe_0.986_I_0.014_ sample: (a) Seebeck coefficient *S*, (b) electrical conductivity *σ*, (c) total thermal conductivity *κ*, and (d) figure-of-merit *zT* as functions of temperature.


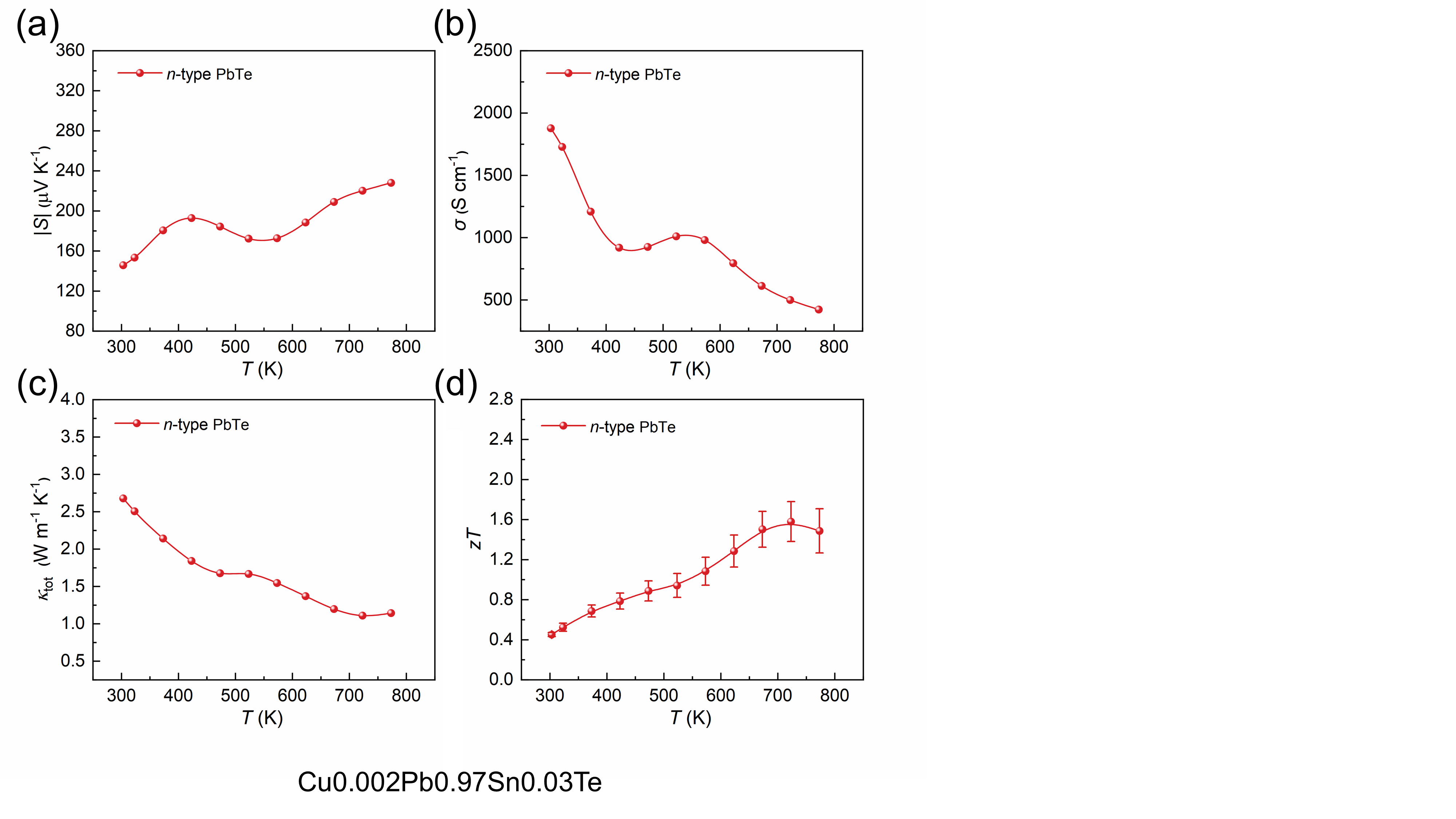


**Figure S15**. Thermoelectric transport properties of *n*-type PbTe (Cu_0.002_Pb_0.97_Sn_0.03_Te) used for single-stage device fabrication: (a) electrical conductivity *σ*, (b) Seebeck coefficient *S*, (c) total thermal conductivity *κ*_total_, and (d) figure-of-merit *zT* as functions of temperature.


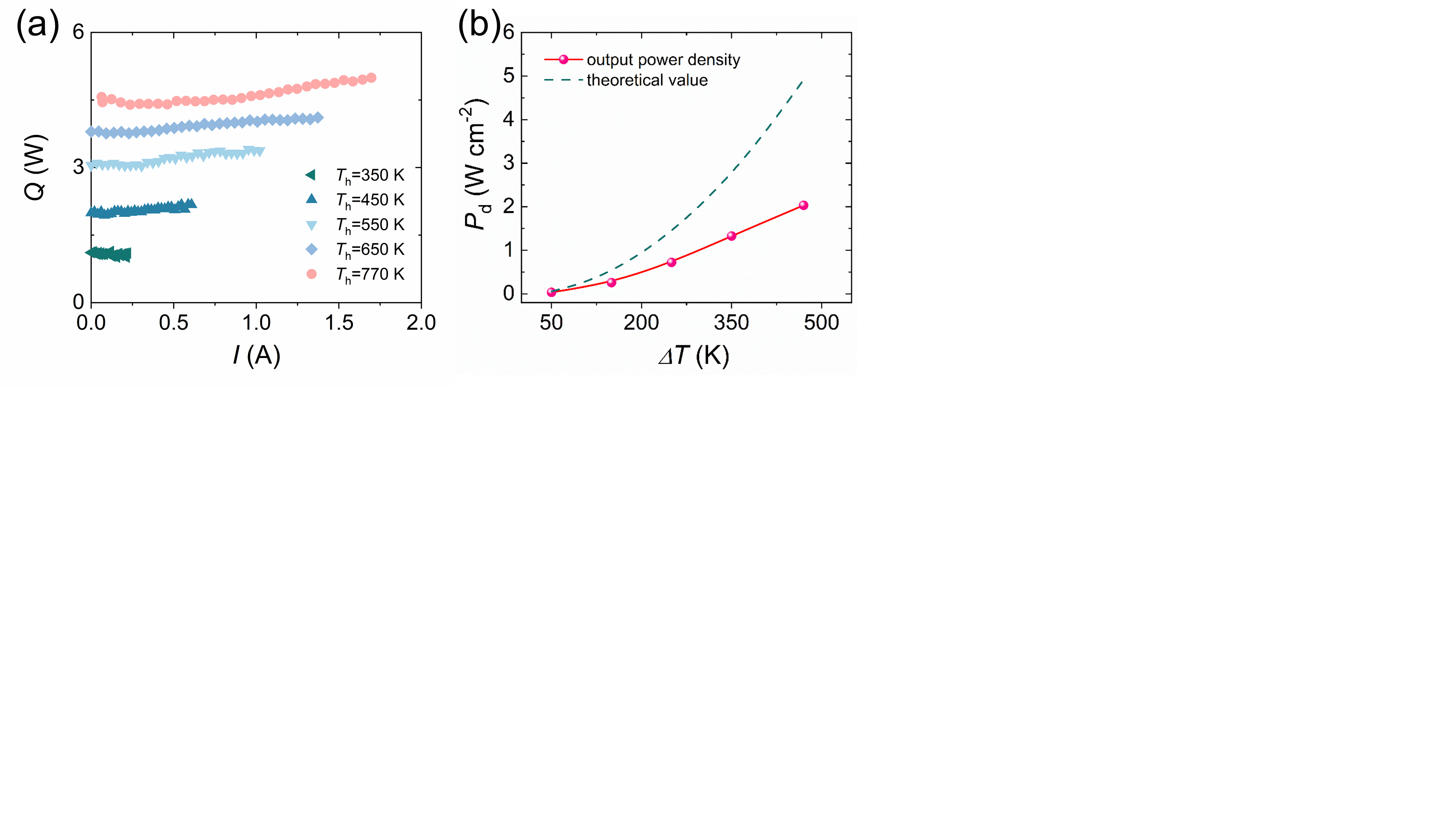


**Figure S16**. Thermoelectric device performance of the 7-pair T_0.986_AGS-I_0.014_/PbTe device. (a) Measured heat flow (*Q*) as a function of the applied temperature difference Δ*T*. (b) Comparison of the predicted output power density (*P*_d_, assuming negligible contact resistance *R*_contant_) and experimentally measured values, with the cold-side temperature fixed at *T*_c_ = 300 K.





**Figure S17**. Thermoelectric cooling performance of the T_0.986_AGS-I_0.014_/commercial *n*-type Bi_2_Te_3_ 7-pair device.^[13-18]^ The maximum temperature difference (Δ*T*_max_) is plotted as a function of the hot-side temperature (*T*_h_).

**Supplementary Tables**

**Table S1. Room-temperature densities of GeTe-based samples with Ag, Sb, and I.**

| **Sample** | ***ρ* (g/cm^3^)** |
| --- | --- |
| GeTe | 6.14 |
| GeTe_0.986_I_0.014_ | 6.13 |
| TAGS | 6.24 |
| T_1-_*_x_*AGS-I*_x_* (*x*=0.006) | 6.33 |
| T_1-_*_x_*AGS-I*_x_* (*x*=0.010) | 6.27 |
| T_1-_*_x_*AGS-I*_x_* (*x*=0.014) | 6.28 |
| T_1-_*_x_*AGS-I*_x_* (*x*=0.018) | 6.25 |

**Table S2. Room-temperature thermoelectric transport properties of the commercial *n*-type Bi_2_Te_3_ sample.**

| **Parameters** | **Values** |
| --- | --- |
| Seebeck coefficient *S* (μV K^-1^) | ~-200 |
| Electrical conductivity *σ* (s cm^-1^) | ~1000 |
| Total thermal conductivity *κ* (W m^-1^ K^-1^) | ~1.42 |
| Figure-of-merit *zT* | ~0.86 |

**Supplementary References**

[1] G. Kresse, J. Furthmüller, *Phys. Rev. B* **1996**, *54*, 11169.

[2] J. Furthmüller, J. Hafner, G. Kresse, *Phys. Rev. B* **1996**, 53, 7334

[3] G. Kresse, J. Furthmüller, *Comp. Mater. Sci.* **1996**, 6, 15.

[4] V. Wang, N. Xu, J.-C. Liu, G. Tang, W.-T. Geng, *Comput. Phys. Commun.* **2021**, 267, 108033.

[5] J. P. Perdew, K. Burke, M. Ernzerhof, *Phys. Rev. Lett.* **1996**, 77, 3865.

[6] Z. Bu, X. Zhang, Y. Hu, Z. Chen, S. Lin, W. Li, Y. Pei, *Energy Environ. Sci.* **2021**, *14*, 6506.

[7] Z. Bu, X. Zhang, B. Shan, J. Tang, H. Liu, Z. Chen, S. Lin, W. Li, Y. Pei, *Sci. Adv.* **2021**, *7*, eabf2738.

[8] S. Zhang, Z. Chen, Q. Bai, W. Li, Y. Pei, *Mater. Today Phys.* **2022**, *26*, 100746.

[9] G. Liang, T. Lyu, L. Hu, W. Qu, S. Zhi, J. Li, Y. Zhang, J. He, J. Li, F. Liu, C. Zhang, W. Ao, H. Xie, H. Wu, *ACS Appl. Mater. Interfaces* **2021**, *13*, 47081.

[10] Y. Jin, Y. Qiu, S. Bai, H. Xie, S. Liu, T. Hong, X. Gao, Y. Wen, L.-D. Zhao, *Adv. Energy Mater.* **2024**, *14*, 2400623.

[11] M. Samanta, T. Ghosh, R. Arora, U. V. Waghmare, K. Biswas, *J. Am. Chem. Soc.* **2019**, *141*, 19505.

[12] H.-S. Kim, P. Dharmaiah, B. Madavali, R. Ott, K.-H. Lee, S.-J. Hong, *Acta Mater.* **2017**, *128*, 43.

[13] S. Wang, S. Bai, P. Chen, S. Zhan, Y. Tian, L. Wang, D. Liu, J. Peng, Y. Li, D. Gao, T. Gao, Z. Zhang, Z. Si, Y. Wei, H. Xie, X. Gao, Y. Zhu, Y. Wen, L.-D. Zhao, *J. Am. Chem. Soc.* **2025**, *147*, 15827.

[14] S. Zhan, S. Bai, B. Qin, Y. Zhu, S. Wang, D. Liu, T. Hong, X. Gao, L. Zheng, Y. Wen, L. D. Zhao, *Adv. Funct. Mater.* **2024**, *34*, 2406428.

[15] S. Liu, Y. Qin, Y. Wen, H. Shi, B. Qin, T. Hong, X. Gao, Q. Cao, C. Chang, L. D. Zhao, *Adv. Funct. Mater.* **2024**, *34*, 2315707.

[16] W. Xu, B. A. Al‐Maythalony, J. Li, X. Li, L. Fu, B. Xu, *Adv. Funct. Mater.* **2024**, *35*, 2414194.

[17] S. Liu, S. Bai, Y. Wen, J. Lou, Y. Jiang, Y. Zhu, D. Liu, Y. Li, H. Shi, S. Liu, L. Wang, J. Zheng, Z. Zhao, Y. Qin, Z. Liu, X. Gao, B. Qin, C. Chang, C. Chang, L.-D. Zhao, *Science* **2025**, *387*, 202.

[18] F. Jiang, C. Lin, J. Cheng, H. Yu, Y. Zhou, X. Ma, L. Wu, S. Ye, J. Chen, S. Zhi, Y. Xu, P. Zhao, X. Wang, F. Cao, Q. Zhang, J. Mao, *Adv. Funct. Mater.* **2024**, *35*, 2415000.
